# A Novel Dried Blood Spot Detection Strategy for Characterizing Cardiovascular Diseases

**DOI:** 10.3389/fcvm.2020.542519

**Published:** 2020-10-09

**Authors:** Linsheng Liu, Xurui Jin, Yangfeng Wu, Mei Yang, Tao Xu, Xianglian Li, Jianhong Ren, Lijing L. Yan

**Affiliations:** ^1^Clinical Pharmacology Research Laboratory, The First Affiliated Hospital of Soochow University, Suzhou, China; ^2^Global Health Research Center, Duke Kunshan University, Kunshan, China; ^3^Peking University Clinical Research Institute, Beijing, China; ^4^Suzhou BioNovoGene Metabolomics Platform, Suzhou, China; ^5^The Key Laboratory of Developmental Genes and Human Disease, Institute of Life Sciences, Southeast University, Nanjing, China; ^6^The Therapeutic Antibody Research Center of SEU-Alphamab, Southeast University, Nanjing, China

**Keywords:** cardiovascular diseases, biomarker, risk prediction, DBS, metabolomics

## Abstract

Cardiovascular diseases (CVDs) are the leading cause of death in China. Conventional diagnostic methods are dependent on advanced instruments, which are expensive, inaccessible, and inconvenient in underdeveloped areas. To build a novel dried blood spot (DBS) detection strategy for imaging CVDs, in this study, a total of 12 compounds, including seven amino acids [homocysteine (Hcy), isoleucine (Ile), leucine (Leu), valine (Val), phenylalanine (Phe), tyrosine (Tyr), and tryptophan (Trp)], three amino acid derivatives [choline, betaine, and trimethylamine N-oxide (TMAO)], L-carnitine, and creatinine, were screened for their ability to identify CVD. A rapid and reliable method was established for the quantitative analysis of the 12 compounds in DBS. A total of 526 CVD patients and 200 healthy volunteers in five provinces of China were recruited and divided into the following groups: stroke, coronary heart disease, diabetes, and high blood pressure. The orthogonal projection to latent structures-discriminant analysis (OPLSDA) model was used to characterize the difference between each CVD group. Marked differences between the groups based on the OPLSDA model were observed. Based on the model, the patients in the three training sets were mostly accurately categorized into the appropriate group. In addition, the receiver operating characteristic (ROC) curves and logistic regression of each metabolite chosen by the OPLSDA model had an excellent predictive value in both the test and validation groups. DBS detection of 12 biomarkers was sensitive and powerful for characterizing different types of CVD. Such differentiation may reduce unnecessary invasive coronary angiography, enhance predictive value, and complement current diagnostic methods.

## Introduction

Cardiovascular diseases (CVDs) and cerebrovascular diseases refer to all heart and cerebral diseases related to vasculopathy, which mainly include coronary heart disease (CHD), hypertension, and stroke (1). Diabetes mellitus is a risk factor for CVD (2). CVD is a serious threat to the health of humans, especially middle-aged and older people over 50 years old, who have a high prevalence, high disability, and high mortality rate. Up to 30 million people die of CVD every year worldwide (3). The age-standardized death rate attributable to all CVDs in the US population was 223.9 per 100,000 (4). Stroke and ischemic heart disease were the leading causes of death in China in 2017 (5). Even when utilizing the most advanced and sophisticated treatments available, more than 50% of survivors are unable to take care of themselves (3).

Studies have shown that the pathogenesis of CVD may be due to blood vessel lesions, which are secretive, gradual, and systemic, and it is difficult to identify obvious clinical symptoms in the early stage (6). Finding reasonable and effective biomarkers to diagnose, classify, and guide the treatment of CVD has always been a focus of clinical diagnostics (1). CVD is essentially a metabolic disease (7). At present, a series of relatively mature and widely clinically used biomarker tests have provided an important reference for the diagnosis and treatment of CVD. The more mature cardiovascular markers mainly include the blood vessels themselves, markers of the coagulation system (such as platelets and fibrinolysis), lipid metabolism and inflammatory markers, plaque calcification, non-calcified detachment predictive markers, and organ damage markers, such as myocardial injury and brain injury markers. However, these diagnostic methods require advanced instruments and are expensive, requiring patients to enter the hospital for examination; additionally, these methods are difficult to fully develop implementing in underdeveloped areas.

Dried blood spot (DBS) technology that can be stored stably enables new possibilities for bioanalytical procedures that can be beneficial for patients, health care providers, and laboratories, and the technology can be inexpensive (8). The sampling can be performed in a non-hospital environment and is suitable for large-scale disease screening (9). DBS technology also reduces the sample processing burden and is characterized by straightforward waste disposal. A DBS method was applied for the detection of metabolites toward hypertension and healthy controls (10). This study aimed to screen and validate CVD biomarkers that can be measured by DBS testing. In this article, we selected 12 biomarkers [choline, betaine, trimethylamine N-oxide (TMAO), creatinine, L-carnitine, homocysteine (Hcy), isoleucine (Ile), leucine (Leu), valine (Val), phenylalanine (Phe), tyrosine (Tyr), tryptophan (Trp)] from the literature that may characterize CVD features. After quantifying the 12 biomarkers in DBSs, the orthogonal projection to latent structures-discriminant analysis (OPLSDA) model was used to screen the potential biomarkers. Then, the receiver operating characteristic (ROC) curve and logistic algorithm were applied to three validation sets to verify whether the selected markers could characterize the occurrence of CVD for clinical diagnosis and risk prediction.

## Materials and Methods

### Biomarker Screening

A literature search was conducted using the PubMed and EMBASE databases, and the Preferred Reporting Items for Systematic Reviews and Meta-Analyses (PRISMA) statement was used (11). The search terms were “cardiovascular diseases” OR “coronary artery disease” OR “stroke” OR “diabetes mellitus” AND “metabolite” OR “metabolomics” OR “biomarkers.” There was no language restriction, and the period covered database inception until October 31, 2017. Our inclusion criteria were as follows: (1) study subjects were humans; (2) study subjects had only metabolic syndrome but no other diseases; and (3) studies reported changes in metabolites in the plasma or serum resulting from metabolic syndrome. To further compile a comprehensive list of relevant literature, manual searches of the reference lists in book chapters and gray literature were conducted. The key metabolites associated with CVD are summarized in Supplementary Table 1. Considering the compatibility of DBS sampling and mass spectrometry analysis, 12 compounds (choline, betaine, TMAO, creatinine, L-carnitine, Hcy, Ile, Leu, Val, Phe, Tyr, Trp) were selected for clinical validation.

### Participants

The study protocol was approved by the Ethics Committee of The First Affiliated Hospital of Soochow University. Male and female adults who were diagnosed with stroke, CHD, diabetes, and high blood pressure (HBP) as well as healthy individuals (HIs) were recruited from five provinces (Hebei, Shaanxi, Liaoning, Ningxia, and Shanxi) in China. Participants fully understood the risks and benefits of the study and provided informed consent. Capillary blood was spotted on paper cards (whatman filter paper) by direct application from the fingertip. According to the severity of the diseases, patients with CVD were divided into four groups: stroke, CHD, diabetes, and HBP. Patients in one group may have also had subsequent complications. That is, if a patient had stroke, diabetes, CHD, and HBP, s/he would be assigned to the stroke group (Table 1).

**Table 1:** Population characteristics in communities study (Five provinces: Hebei, Shaanxi, Liaoning, Ningxia, and Shanxi).

	**HI (*n* = 200)**	**Stroke (*n* = 151)**	**CHD (*n* = 173)**	**Diabetes (*n* = 105)**	**HBP (*n* = 97)**
Mean age, years (SD)	64.5 (11.3)	64.6 (7.8)	65.4 (8.9)	64.1 (6.9)	59.3 (11.9)
**Sex**					
Men	100 (50%)	89 (58.9%)	84 (48.6%)	52 (49.5%)	47 (48.5%)
Women	100 (50%)	62 (41.1%)	89 (51.4%)	53 (50.5%)	50 (51.5%)
Mean BMI, kg/m^2^ (SD)	23.2 (3.5)	25.3 (3.4)**	25.6 (4.1)**	24.8 (3.6)**	25.6 (3.6)**
DBP (SD)	75.4 (8.2)	90.7 (17.2)**	89.9 (15.4)**	86.6 (14.7)**	93.5 (13.8)**
**Complication**					
CHD, (Unknown)^#^	NA	26, 17.2% (2, 1.3%)	NA	NA	NA
Diabetes, (Unknown)^#^	NA	7, 4.6% (5, 3.3%)	7, 4.0% (6, 3.4%)	NA	NA
Hypertension	NA	133 (88.1%)	126 (72.8%)	64 (61.0%)	NA
Use therapeutic drugs within a month	NA	120 (79.5%)	128 (74.0%)	70 (66.7%)	32 (27.8%)
**Smoking**					
Current smoker	74 (37.0%)	27 (17.9%)	40 (23.1%)	22 (21.0%)	25 (25.8%)
Never smoker	126 (63.0%)	124 (82.1%)	133 (76.9%)	83 (79.0%)	72 (74.2%)
Drinking	26 (13.0%)	12 (7.9%)	18 (10.4%)	7 (6.7%)	21 (21.6%)
Farm work	140 (70.0%)	62 (41.1%)	95 (54.9%)	52 (49.5%)	64 (66.0%)
Education attainment, years (SD)	6.5 (3.6)	5.3 (3.4)	4.3 (3.6)	5.1 (3.6)	4.9 (3.5)

# *Some missing values for this category. NA = not applicable. BMI = body-mass index*.

** *P < 0.01 vs. HI*.

*HI, Healthy individuals; CHD, Coronary Heart Disease; HBP, High Blood Pressure*.

### Dried Blood Spot Extraction and Methodology Validation

For preparation of calibration curves and quality control (QC) of the metabolites, DBSs spiked with known concentrations were used. The concentration ranges of 12 metabolites are shown in Table 2. Six-millimeter (diameter) DBS was placed in a 96-well protein precipitation plate, 20 μl of 10 mg/ml dithiothreitol (DTT) was added to each well, and the plate was shaken at 600 rpm for 5 min. Then, 300 μl mixed extracting solution [0.1% formic acid–acetonitrile containing 5 μl internal standard (IS)] was added to each well and shaken at 600 rpm for 30 min. The extraction solution was filtered by 0.02 MPa and collected. Each sample was diluted with 200 μl of 80% (V/V) acetonitrile water solution and mixed at 600 rpm for 5 min. All of the calibration curves were computed by plotting the relative peak area ratios of analyte to IS vs. the plasma concentrations of each analyte using a weight factor of 1/x^2^ at each concentration.

**Table 2:** MS/MS detection parameters and calibration curves of 12 compound with the internal standard.

**Target**	**Precursor/product ion**	**DP**	**EP**	**CE**	**CXP**	**Regression equation**	**Correlation (r)**	**Accuracy**	**Linear range (μmol/L)**
Betaine	118.1/41.9	56	10	75	2	*y* = 0.0174x + 0.0259	0.9928	94.5~109%	5~400
Choline	103.8/60.0	101	10	25	12	*y* = 0.0435x – 0.00316	0.9982	91.7~106%	0.5~40
TMAO	76.0/58.0	41	10	27	6	*y* = 1.55x – 0.021	0.9988	97.7~104%	0.25~20
Creatinine	114.0/86.1	46	10	17	16	*y* = 0.0178x + 0.00184	0.9983	96.2~109%	2.5~200
L-Carnitine	162.0/59.9	61	10	29	10	*y* = 0.116x + 0.0277	0.9961	92.3~113%	1~100
Hcy	136.0/90	46	10	15	8	*y* = 0.121x – 0.000856	0.9967	90.7~104%	0.5~40
Ile	132.0/69.00	51	10	25	6	*y* = 9453x + 3323	0.9983	94.3~104%	2~200
Leu	132.0/43.00	56	10	35	2	*y* = 2.1703x + 2.2E03	0.9976	94.3~104%	2~200
Val	118.2/72.000	26	10	15	3	*y* = 0.0406x + 0.0583	0.9947	96.8~107%	10~500
Phe	166.1/119.9	46	10	19	10	*y* = 0.112x + 0.067	0.9942	94.9~108%	2.5~200
Tyr	182.1/165.2	46	10	13	16	*y* = 19804x + 402	0.9983	94.3~104%	2~200
Trp	205.1/188.1	31	10	15	16	*y* = 64904x – 2643	0.9987	95.0~105%	2.5~200
Choline d9^#^	113.0/69.0	26	10	25	6				
Betaine d9^#^	127.1/68.0	66	10	27	4				
TMAO d9^#^	85.0/66.0	41	10	29	12				
Creatinine d3^#^	117.0/88.9	61	10	11	8				
L-Carnitine d3^#^	165.1/103.1	56	10	23	10				
Val-C13^#^	119.0/72.0	41	10	15	6				
Phe-C13^#^	167.1/120.1	41	10	19	12				
Hcy-d4^#^	140.0/94.00	36	10	17	2				

# *Internal Standard*.

*DP, Declustering Potential; EP, Entrance Potential; CE, Collision Energy; CXP, Collision Cell Exit Potential*.

### Data Acquisition

Ultra-performance liquid chromatography (UPLC) separation was performed on a Waters ACQUITY UPLC® BEH HILIC column (2.1 × 100 mm, 1.7 μm) at 40°C at a flow rate of 0.4 ml/min. The autosampler was conditioned at 4°C, and the injection volume was 5 μl. The two mobile phases consisted of 0.1% formic acid−10 mmol/L ammonium formate in water (solvent A) and acetonitrile (solvent B). Separation was carried out in 5 min under the following conditions: 0~1 min, 80% B; 1~2 min, 80~70% B; 2~2.5 min, 70% B; 2.5~3 min, 70~80% B; and 3~5 min, 80% B.

An API 4000 mass spectrometer equipped with an electrospray ionization (ESI) source (AB SCIEX, USA) was used to acquire mass spectra profiles. The optimized operating parameters were as follows: source voltage, 5.0 kV (positive mode); and curtain gas (CUR), 30 psi. Quantitation was performed using MRM mode to monitor the protonated precursor to product ion transition. The compound-dependent parameters, such as m/z, declustering potential (DP), focusing potential (FP), collision energy (CE), and cell exit potential (CXP) were optimized and are shown in Table 2.

### Logistic Algorithm and Statistical Analysis

MS data were processed using AB SCIEX Analyst 1.6 software, and compound concentrations of DBS samples were calculated. Compound concentrations of DBS samples, participant characteristics [body mass index (BMI), age, diastolic blood pressure (DBP), and sex], and clinical diagnoses were analyzed using the logistic algorithm based on the genetic algorithm to fit the grid model. To verify model accuracy and hopefully national promotion, data from 358 participants in two provinces (test phase, Hebei and Shaanxi) were analyzed as a training set for building the model, and data from 368 participants in the other three provinces (Liaoning, Ningxia, and Shanxi) were used for model validation to verify the accuracy of the model.

OPLSDA was applied to explore the differences in metabolic profiles between each group to identify the response variables that contributed most strongly to the model (SIMCA version 13.0 Umetrics AB, Umea, Sweden). The model was evaluated using three quantitative parameters: R2X is the explained variation in X, R2Y is the explained variation in Y, and Q2 is the predicted variation in Y. The values of Q2 approaching one indicate the perfect fit of the model. Variable importance in the projection (VIP) >1 contributed most to the model and the prediction. In addition, one-way ANOVA with Bonferroni and Hochberg correction was applied for each metabolite. Heat maps were generated using R GUI after standardization between groups (Supplementary Figure 1). ROC analysis and logistic regression analysis were performed to differentiate different CVD categories. The cutoff value was calculated by maximizing Yoden's index with equal weighting for sensitivity and specificity. Statistical analyses were performed using SPSS software version 19.0 (IBM Corp., Armonk, New York). A *p*-value of <0.05 was considered statistically significant.

## Results

### Participant Characteristics

In this study, the 726 enrolled participants (Table 1) belonged to five groups: no CVD or other diseases (HI group, *n* = 200), stroke (*n* = 151), CHD (*n* = 173), diabetes (*n* = 105), and HBP (*n* = 97). The distributions of sex and age among the CVD groups and healthy group were not significantly different. The BMI and DBP levels of CVD patients were significantly higher than those of the healthy volunteers. This result was consistent with previous reports, as BMI and DBP are high risk factors for CVD (2, 12, 13).

The training set included 358 participants from Hebei and Shaanxi (96 HI, 74 stroke, 73 CHD, 65 diabetes, and 50 HBP volunteers). Their characteristics are shown in Supplementary Table 2. Compared with HI subjects, CVD patients had higher levels of BMI and HBP and a low proportion of smoking and farm work. Interestingly, the higher the disease risk, the greater the percentage of medication use found.

The validation sets from Liaoning, Ningxia, and Shanxi included 368 participants. Their demographic information is shown in Supplementary Tables 3–5. The higher levels of BMI and HBP and the low proportion of smoking and farm work of CVD patients found in the test phase were also found in these three data sets.

### Method Validation

The method was validated for linearity, extraction recovery, accuracy, and precision in this study. Detailed results of the methodology validation are provided in Table 2 and Supplementary Tables 6, 7.

### Cross-Comparisons Between and Within Cardiovascular Disease Groups

**Figure 1:**
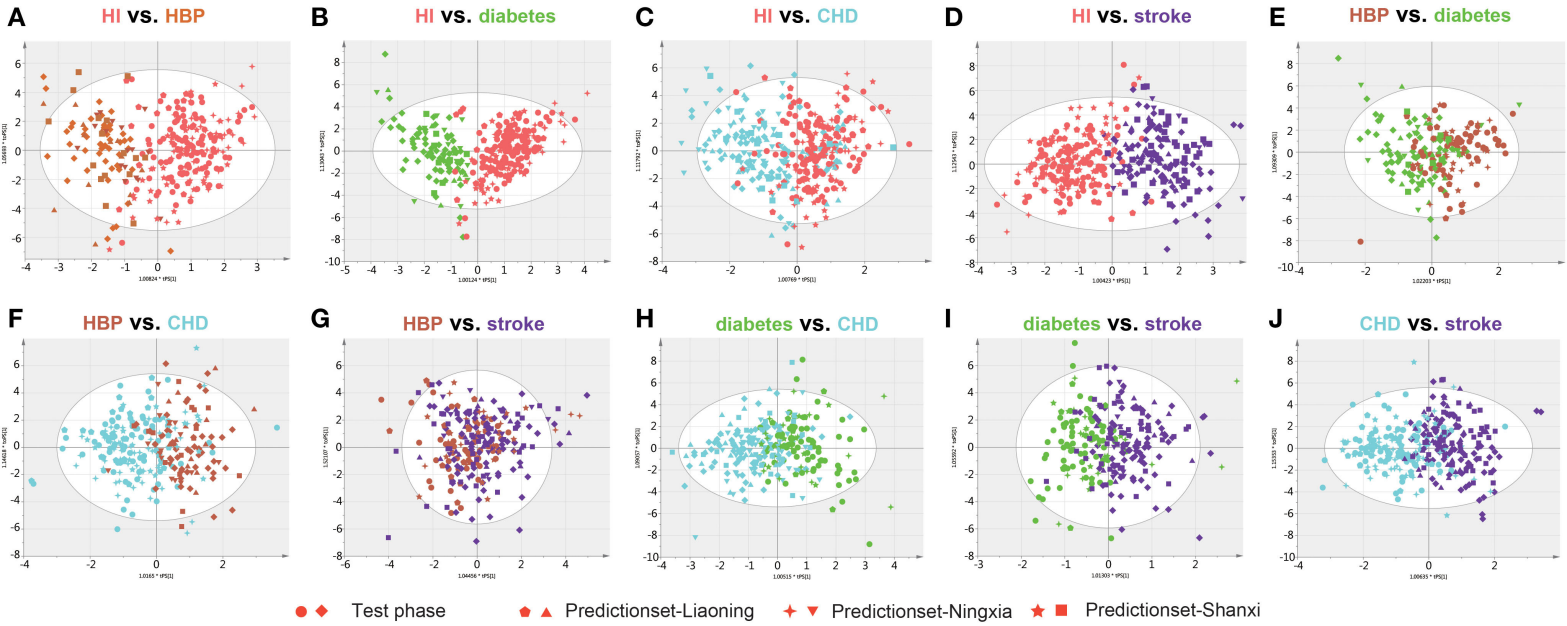
Orthogonal projection to latent structures-discriminant analysis (OPLSDA) prediction modeling to characterize the difference between each cardiovascular disease (CVD) group using the test samples with the 12 metabolites **(A–J)**. There were obvious differences between the groups in the OPLSDA model. According to this prediction model, individuals in the three training groups could be categorized into the correct region of the prediction set. HI, healthy individual; CHD, coronary heart disease; HBP, high blood pressure.

Twelve biomarkers including amino acids, amino acid derivatives, L-carnitine, and creatinine were measured in DBS samples. The OPLSDA model was used to characterize the difference between each CVD group and to find discrepant compounds *via* variable importance in the projection (VIP). We also validated the patient classification in the three training groups by establishing the OPLSDA model. There were obvious differences between the groups in the OPLSDA model (Figure 1), which provides a theoretical basis for us to establish a prediction model. In Figure 1, we can see that according to the established OPLSDA model, the patients in the three training groups were mostly categorized into the appropriate group. The metabolites with VIP values >1.0 were considered potential differential metabolites. Individuals with various types of CVD were compared to HI and with each other, characterizing specific metabolites.

Clear differences were identified, and the three quantitative parameters are shown in Supplementary Table 8. The metabolites with VIP values >1.0 are shown in Table 3. Concentration changes associated with Hcy, Lle, Ile, Trp, and creatinine were the most significant in all paired comparisons.

**Table 3:** Statistical analysis of diagnostic biomarkers: discovery phase.

**Metabolites**	**VIP value**	**Fold change**	*p*-value*	***p*-value^#^**
**HI vs. HBP**				
Hcy	1.86	1.27	<0.0001	<0.0001
Trp	1.71	0.61	<0.0001	<0.0001
Leu	1.47	1.38	<0.0001	<0.0001
**HI vs. Diabetes**				
Trp	1.80	0.51	<0.0001	<0.0001
Leu	1.39	1.53	<0.0001	<0.0001
Hcy	1.30	1.18	<0.0001	<0.0001
**HI vs. CHD**				
Trp	1.81	0.76	<0.0001	<0.0001
Leu	1.25	1.16	<0.0001	<0.0001
TMAO	1.23	1.39	0.0002	0.0002
Creatinine	1.15	1.12	0.0556	0.0540
**HI vs. Stroke**				
Hcy	1.68	1.26	<0.0001	<0.0001
Leu	1.43	1.45	<0.0001	<0.0001
Trp	1.38	0.70	<0.0001	<0.0001
**HBP vs. Diabetes**				
Trp	1.74	0.84	0.337	0.228
Hcy	1.54	0.93	0.0021	<0.0001
TMAO	1.29	1.53	0.0144	0.0143
Val	1.19	1.13	0.0856	0.0821
Creatinine	1.09	1.17	0.0811	0.0779
**HBP vs. CHD**				
Hcy	2.20	0.78	<0.0001	<0.0001
Leu	1.25	0.84	0.0015	<0.0001
Trp	1.12	1.25	0.0105	0.0104
Creatinine	1.06	1.14	0.2167	0.1958
**HBP vs. Stroke**				
Creatinine	1.84	1.19	0.0258	0.0255
Trp	1.51	1.14	0.5432	0.4256
L-Carnitine	1.48	1.20	0.0380	0.0373
**Diabetes vs. CHD**				
Trp	1.66	1.49	<0.0001	<0.0001
Hcy	1.51	0.84	<0.0001	<0.0001
Leu	1.48	0.76	0.029	0.028
Val	1.05	0.86	0.0075	0.075
**Diabetes vs. Stroke**				
Trp	2.62	1.41	<0.0001	<0.0001
Hcy	1.37	1.09	0.0002	<0.0001
L-Carnitine	1.06	1.14	0.0749	0.0722
**CHD vs. Stroke**				
Hcy	2.19	1.28	<0.0001	<0.0001
Leu	1.47	1.25	<0.0001	<0.0001

*VIP, Variable Importance for the projection of OPLSDA model; HI, Healthy individuals; CHD, Coronary Heart Disease; HBP, High Blood Pressure*.

* *One-way ANOVA with Bonferroni correction*.

# *One-way ANOVA with Hochberg correction*.

### Differential Metabolites and Diagnosis of Cardiovascular Disease via Dried Blood Spots

Through 10 comprehensive cross-comparisons of different groups, seven differential metabolites were confirmed. The concentrations of all the metabolites in HI and individuals in the different CVD groups are summarized in Table 3 and Supplementary Tables 9–11.

**Figure 2:**
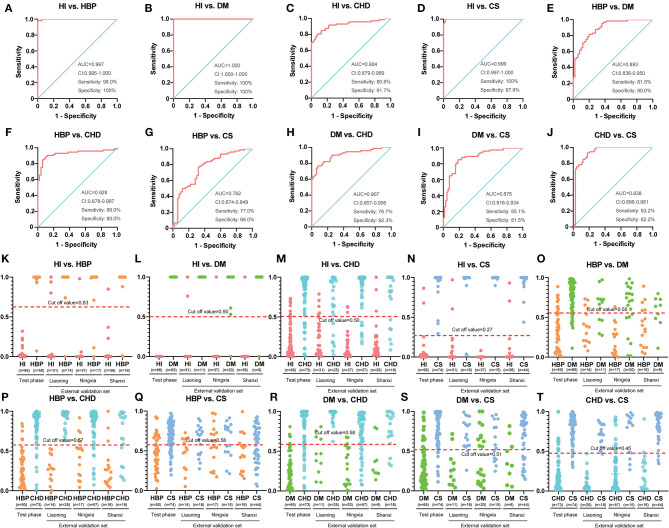
Diagnostic outcomes and prediction accuracies with population characteristics. **(A–J)** The receiver operating characteristic (ROC) curves, on the basis of the logistic regression of each metabolite from the test set. **(K–T)** The prediction accuracies by the biomarkers in the test phase and validation sets were compared between each group. HI, healthy individual; DM, diabetes mellitus; CHD, coronary heart disease; HBP, high blood pressure.

As summarized in Table 3, the accurate diagnosis of different types of CVD is fundamental for precision medicine. The criteria for biomarkers were VIP >1.0 in each OPLSDA model was used for differential diagnosis. The ROC curves, on the basis of the logistic regression of each metabolite from the test set, are displayed in Supplementary Figure 2; the areas under the curve (AUCs), sensitivity, and specificity were used to evaluate the prediction accuracy. The patient characteristics, including BMI, age, gender, and DBP, were also taken into consideration. Although it has no effect in many models, it still improves the AUC for a few models, especially in HBP vs. stroke (0.762 vs. 0.653; Figure 2, Supplementary Figure 2). This means that patient characteristics partially characterize CVD.

The AUC, sensitivity, and specificity were 0.997, 98.0%, and 100% for HI vs. HBP (Figure 2A); 1.000, 100%, and 100% for HBP vs. diabetes (Figure 2B); 0.924, 80.8%, and 91.7% for HI vs. CHD (Figure 2C); 0.999, 100%, and 97.9% for HI vs. stroke (Figure 2D); 0.893, 81.5%, and 80.0% for HBP vs. diabetes (Figure 2E); 0.928, 89.0%, and 90.0% for HBP vs. CHD (Figure 2F); 0.762, 77.0%, and 68.0% for HBP vs. stroke (Figure 2G); 0.907, 76.7%, and 92.3% for diabetes vs. CHD (Figure 2H); 0.875, 85.1%, and 81.5% for diabetes vs. stroke (Figure 2I); and 0.938, 93.2%, and 82.2% for CHD vs. stroke (Figure 2J); respectively. For additional cross-comparisons, AUCs ranged from 0.519 to 1.000, sensitivities from 52.3 to 100%, and specificities from 35.3 to 100% in the other three-center external validation sets, which are shown in Supplementary Figures 3–8. The logistic regression curve and cutoff values in all cross-comparisons in the test phase are shown in Supplementary Tables 12, 13.

Based on the highest prediction sensitivity and specificity of the ROC in the test phase, the optimal cutoff values were 0.63 for HI vs. HBP (Figure 2K), 0.50 for HI vs. diabetes and HI vs. CHD (Figures 2L,M), 0.27 for HI vs. stroke (Figure 2N), 0.55 for HBP vs. diabetes (Figure 2O), 0.57 for HBP vs. CHD (Figure 2P), 0.58 for HBP vs. stroke and diabetes vs. CHD (Figures 2Q,R), 0.51 for diabetes vs. stroke (Figure 2S), and 0.45 for CHD vs. stroke (Figure 2T). The cutoff values were then used to predict the different types of CVD in the test phase and external sets. The predictive accuracy was 100% for HI vs. HBP, HI vs. diabetes and HI vs. stroke, 86.4% for HI vs. CHD, 79.1% for HBP vs. diabetes, 89.4% for HBP vs. CHD, 72.6% for HBP vs. stroke, 84.1% for diabetes vs. CHD, 83.5% for diabetes vs. stroke, and 87.8% for CHD cs. stroke in the test phase (Table 4). The predictive accuracy of the three-centered external validation sets was shown in Supplementary Tables 14–20, and other results of the laboratory diagnostic evaluation indicators obtained by the compounds we selected are shown in Table 4 and Supplementary Tables 14–20.

**Table 4:** Diagnostic test evaluation index of our model in the test phase with population characteristics.

**Group**	**Sensibility**	**Specificity**	**Accuracy**	**TPF**	**FPF**	**PPV**	**NPV**
HI vs. HBP	98.0%	100.0%	100.0%	0.0%	2.0%	100.0%	100.0%
HI vs. Diabetes	100.0%	100.0%	100.0%	0.0%	0.0%	100.0%	100.0%
HI vs. CHD	80.7%	91.7%	86.4%	8.3%	19.3%	86.8%	78.4%
HI vs. Stroke	100.0%	97.9%	100.0%	2.1%	0.0%	100.0%	100.0%
HBP vs. Diabetes	81.5%	80.0%	79.1%	20.0%	20.0%	81.5%	76.0%
HBP vs. CHD	89.0%	90.0%	89.4%	10.0%	11.0%	92.9%	84.9%
HBP vs. Stroke	77.0%	68.0%	72.6%	32.0%	23.0%	77.0%	66.0%
Diabetes vs. CHD	76.7%	92.3%	84.1%	7.7%	23.3%	91.8%	77.9%
Diabetes vs. Stroke	85.1%	81.5%	83.5%	18.5%	14.9%	84.0%	82.8%
CHD vs. Stroke	93.2%	82.2%	87.8%	17.8%	6.8%	84.1%	92.3%

*TPF, true positive fraction; FPF, false positive fraction; PPV, positive predictive value; NPV, negative predictive value; HI, Healthy individuals; CHD, Coronary Heart Disease; HBP, High Blood Pressure*.

As shown in Table 5, there was a difference in the concentration of the 12 metabolites between each group. For example, the level of Trp is the highest in the HI group, while Leu is the lowest among the HI group. After stepwise regression, we found multi-collinearity between Leu and Ile and deleted Ile in subsequent studies. The calculated value can be obtained through our calculation equation (Supplementary Tables 12, 13). After comparing with the cutoff value, high PPV and NPV values can be obtained (Table 4); in other words, different groups could be characterized.

**Table 5:** Concentrations (μmol/L) of 12 differential metabolites in the test phase.

**Metabolites**	**HI (*n* = 96)**	**HBP (*n* = 50)**	**Diabetes (*n* = 65)**	**CHD (*n* = 73)**	**Stroke (*n* = 74)**	***p*-value for trend**
	**Mean ± SD**	**Mean ± SD**	**Mean ± SD**	**Mean ± SD**	**Mean ± SD**	
Betaine	88.2 ± 36.8	79.5 ± 33.8	76.0 ± 32.7	79.5 ± 21.6	86.0 ± 28.8	0.092
Choline	27.2 ± 9.8	33.3 ± 18.5	34.6 ± 18.8	28.9 ± 9.4	32.9 ± 13.9	0.003
TMAO	1.3 ± 0.9	1.4 ± 1.1	2.1 ± 1.8	1.8 ± 1.1	1.7 ± 1.2	<0.001
Creatinine	48.7 ± 11.3	47.9 ± 13.0	55.9 ± 23.2	54.6 ± 13.8	56.8 ± 17.2	<0.001
L-Carnitine	29.4 ± 9.0	27.9 ± 10.8	28.7 ± 10.4	30.6 ± 8.5	33.5 ± 13.5	0.021
Hcy	20.1 ± 3.1	25.4 ± 2.9	23.7 ± 2.8	19.8 ± 5.7	25.4 ± 3.6	<0.001
Ile	25.8 ± 7.5	37.0 ± 10.8	41.1 ± 14.6	30.6 ± 9.6	38.8 ± 12.3	<0.001
Val	152.0 ± 38.1	166.9 ± 44.3	189.1 ± 52.6	163.3 ± 38.3	181.7 ± 49.9	<0.001
Leu	89.3 ± 24.6	123.2 ± 35.1	136.5 ± 46.9	103.7 ± 31.3	129.6 ± 39.9	<0.001
Phe	51.0 ± 13.0	53.5 ± 13.6	57.2 ± 15.6	53.6 ± 14.3	57.1 ± 17.3	0.037
Tyr	43.3 ± 11.1	47.6 ± 14.5	50.6 ± 15.6	48.0 ± 13.7	52.2 ± 19.6	0.002
Trp	42.1 ± 12.1	25.6 ± 7.2	21.5 ± 5.3	31.9 ± 14.2	29.3 ± 8.5	<0.001

*HI, Healthy individuals; CHD, Coronary Heart Disease; HBP, High Blood Pressure*.

## Discussion

As a sensitive and effective technology, DBS was first used for newborn screening (14–16), then it is widely successfully applied to the diagnosis of other diseases (17, 18). Our work describes a targeted metabolomics assessment of 526 patients with CVD and 200 healthy volunteers in five provinces of China. Models constructed from the 12 metabolites showed significant pattern differences between healthy people and patients and between patients of all types. There were at least three significantly regulated metabolites in the DBS samples in each group. The combination of metabolic biomarkers provides excellent predictive value for distinguishing between each disease type and healthy people and those with disease.

Common risk factors for CVD include serum lipids (specifically including total cholesterol, triglycerides, and apolipoproteins), as well as C-reactive protein (CRP) and Hcy (19). Hcy is a degradation product of the process of protein metabolism. Under normal circumstances, Hcy in the blood participates in the body's transsulfuration and transmethylation processes with the aid of enzymes and vitamin B6 and folic acid, is degraded to cysteine, and converted into partial proteins (20). When metabolic disturbances occur, if Hcy cannot be degraded, it will accumulate in the body. A high concentration of Hcy can cause damage to the inner wall of the blood vessel and thickening and roughening of the intima of the blood vessel, forming plaque, which can narrow or even block the lumen, resulting in insufficient blood supply to the artery being incomplete, which leads to atherosclerosis and CHD (21). Numerous studies have shown that hyperhomocysteinemia is an independent risk factor for CVD (22).

The intestinal microbiota metabolism of L-carnitine and choline promotes the development of CVD (23–25). Intestinal microorganisms can use excessive amounts of choline, betaine, and L-carnitine as sources of carbon energy, and their unique trimethylamine lyase can break CN bonds. Trimethylamine (TMA) is released as a metabolic waste product and enters the liver through the portal vein. TMA is further oxidized by the liver's secreted flavin-containing monooxygenase 3 (FMO3) to form TMAO (26). TMAO is associated with cholesterol metabolism, insulin resistance, platelet aggregation, thrombosis, vascular inflammatory response, and atherosclerotic plaque formation, which may lead to atherosclerosis, heart failure, hypertension, and stroke. Gut microbiota-derived TMAO is emerging as a new potentially important cause of increased cardiovascular risk (27, 28).

Creatinine is a product of muscle metabolism in the human body. Its level is related to renal function. Long-term hypertension or heart failure can cause kidney damage, which in turn increases creatinine levels (29).

Val, Leu, and Ile are known as branched-chain amino acids (BCAAs). Elevated BCAAs are associated with numerous systemic diseases, including cancer, diabetes, and heart failure (30, 31). Elevated levels of BCAAs activate mammalian target of rapamycin complex 1 (mTORC1) and downstream p70 ribosomal S6 kinase (S6K1), which block the insulin signaling pathway by inducing insulin receptor substrate 1 (IRS-1) serine phosphorylation, causing insulin resistance (32). High levels of BCAAs may also travel to skeletal muscle, interfere with the accumulation of lipid metabolites, and cause skeletal muscle insulin resistance when degraded in the muscle (30). BCAAs drive vascular fatty acid transport and cause insulin resistance (33). Insulin resistance is an independent and important risk factor for CVD. Insulin resistance aggravates CVD by causing the occurrence and development of abnormal glucose metabolism, impacting lipid metabolism, decreasing nitric oxide (NO) production, inducting hypertension, and reducing fibrinolytic activity (34).

During the development of CVD, interferon-γ-mediated inflammation accelerates the degradation of Trp into downstream metabolites (35). Trp could be catalyzed by an alternative inducible indoleamine-pyrrole 2,3-dioxygenase under certain pathophysiological conditions, such as CVD, which consequently increases the formation of kynurenine metabolites. The kynurenine pathway plays a key role in the increased prevalence of CVD (36), and we also found that individuals in the CVD group had a lower level of Trp than in the healthy subjects in this study (Table 5 and Supplementary Tables 9–11). In addition, clinical studies have revealed that Phe and Tyr metabolic abnormalities are also closely related to CVD (35, 37, 38). However, no differences were found between the groups in our study.

In the ROC model we established, most sensitivity and specificity values were high. Only the HBP and stroke groups were poorly classified: sensitivity and specificity were 77.0% and 68.0% in the test phase, respectively. Since 90.5% of stroke patients enrolled in our study also had HBP, we speculate that it is difficult to distinguish between the two groups.

DBS sampling as a microsampling technology has gained interest for many new applications. Increased interest in DBS technology in various fields enables new possibilities in bioanalytical procedures that can be beneficial for patients, health care providers, and laboratories (8). Very large potential from an economical point of view was also shown for DBS technology. The cost for collecting a DBS sample was estimated to be only 20–25% of that of a conventional venous blood sample (8). Moreover, DBS sampling can be performed in a non-hospital environment with a finger or heel prick by a technician or by the patient himself or herself after training. Less reactivity of analytes was found because the adsorption and drying of blood on a solid phase facilitated shipment and storage and reduced costs. Most pathogenic factors are inactivated during blood adsorption and drying, resulting in safer sample handling (39). At present, there are many methods for the clinical diagnosis of CVD, such as computerized tomography (CT), coronary angiography (40), and image-based cardiac diagnosis with machine learning (41). However, in remote rural areas, these methods are relatively lacking and difficult to implement. Blood sampling is relatively simple and convenient but often difficult to transport and preserve. We have developed a sensitive and powerful DBS detection and logistic algorithm, and better sample stability could be observed. Samples that can be stored at room temperature and the DBS method allow for more convenient blood collection, easier transportation, lower detection cost were also considered, and high forecast accuracy. Convenient and inexpensive multidimensional diagnostic methods are important for people, especially in relatively isolated rural areas and are suitable for clinical diagnosis in areas with undeveloped medical facilities.

### Study Limitations

First, the cardiovascular risk models built in this paper remain incomplete. Analyses of more compounds, such as unsaturated fatty acids, are recommended in future studies. Second, our study population consisted of middle-aged to elderly patients in five provinces of China. Younger age groups with suspected or confirmed CVD could be considered. Third, our strategy requires a mass spectrometry facility, which is not common in some underdeveloped regions, and the equipment is very expensive. Transport of DBS to a qualified laboratory for testing is required in future studies; the scope could be broadened to include other provinces and other ethnicities within Asia, other races, and larger sized samples.

## Conclusions

We analyzed 12 biomarkers from DBSs to characterize CVD features. This improved evaluation can lead to the clinical diagnosis of CVD. Novel algorithms predict and differentiate between CVD types; such differentiation may reduce unnecessary invasive coronary angiography, enhance predictive value, and complement current diagnostic methods.

## Data Availability Statement

All datasets generated for this study are included in the article/Supplementary Material.

## Ethics Statement

The studies involving human participants were reviewed and approved by the study protocol was approved by the Ethics Committee of The First Affiliated Hospital of Soochow University. The patients/participants provided their written informed consent to participate in this study.

## Author Contributions

LL, JR, and LY designed the study. LL, YW, MY, TX, and XL performed experiments and collected and analyzed the data. LL wrote the manuscript. All authors contributed to the article and approved the submitted version.

## Conflict of Interest

MY and JR were employed by the company Suzhou BioNovoGene Metabolomics Platform. The remaining authors declare that the research was conducted in the absence of any commercial or financial relationships that could be construed as a potential conflict of interest.

## Abbreviations

CVD: cardiovascular disease
CHD: coronary heart disease
DBS: dried blood spot
ROC: receiver operating characteristic
TMAO: trimethylamine N-oxide
Hcy: homocysteine
Ile: isoleucine
Leu: leucine
Val: valine
Phe: phenylalanine
Tyr: tyrosine
Trp: tryptophan
HI: healthy individual
HBP: high blood pressure
BMI: body mass index
DBP: diastolic blood pressure
OPLSDA: orthogonal projection to latent structures-discriminant analysis
VIP: variable importance in the projection.
