# Supplementary material for: A Novel Dried Blood Spot Detection Strategy for Characterizing Cardiovascular Diseases
Source: Front Cardiovasc Med. 2020 Oct 9;7:542519. doi: 10.3389/fcvm.2020.542519 (PMC7583634; doi:10.3389/fcvm.2020.542519)
Supplement: Supplementary file 1 [file Data_Sheet_1.docx]

**A Novel Dried Blood Spots Detection Strategy for Characterizing Cardiovascular Diseases**

Linsheng Liu^1^*, Xurui Jin^2^, Yangfeng Wu^3^, Mei Yang^6^, Tao Xu^4,5^, Xianglian Li^1^, Jianhong Ren*^,6^, Lijing L. Yan*^,2^
^1^ Clinical Pharmacology Research Laboratory, The First Affiliated Hospital of Soochow University, Suzhou, Jiangsu, China

^2^ Global Health Research Center, Duke Kunshan University, Kunshan, Jiangsu, China

^3^ Peking University Clinical Research Institute, Beijing, China.

^4^ The Key Laboratory of Developmental Genes and Human Disease, Institute of Life Sciences, Southeast University, Nanjing, Jiangsu, China

^5^ The Therapeutic Antibody Research Center of SEU-Alphamab, Southeast University, Nanjing, China

^6^ Suzhou BioNovoGene Metabolomics Platform, Suzhou, China

*Corresponding author.

E-mail addresses: lijing.yan@dukekunshan.edu.cn (L. Yan), rjh@bionovogene.com (J. Ren).

liulinsheng@suda.edu.cn, linsheng_li@126.com(L. Liu)

**
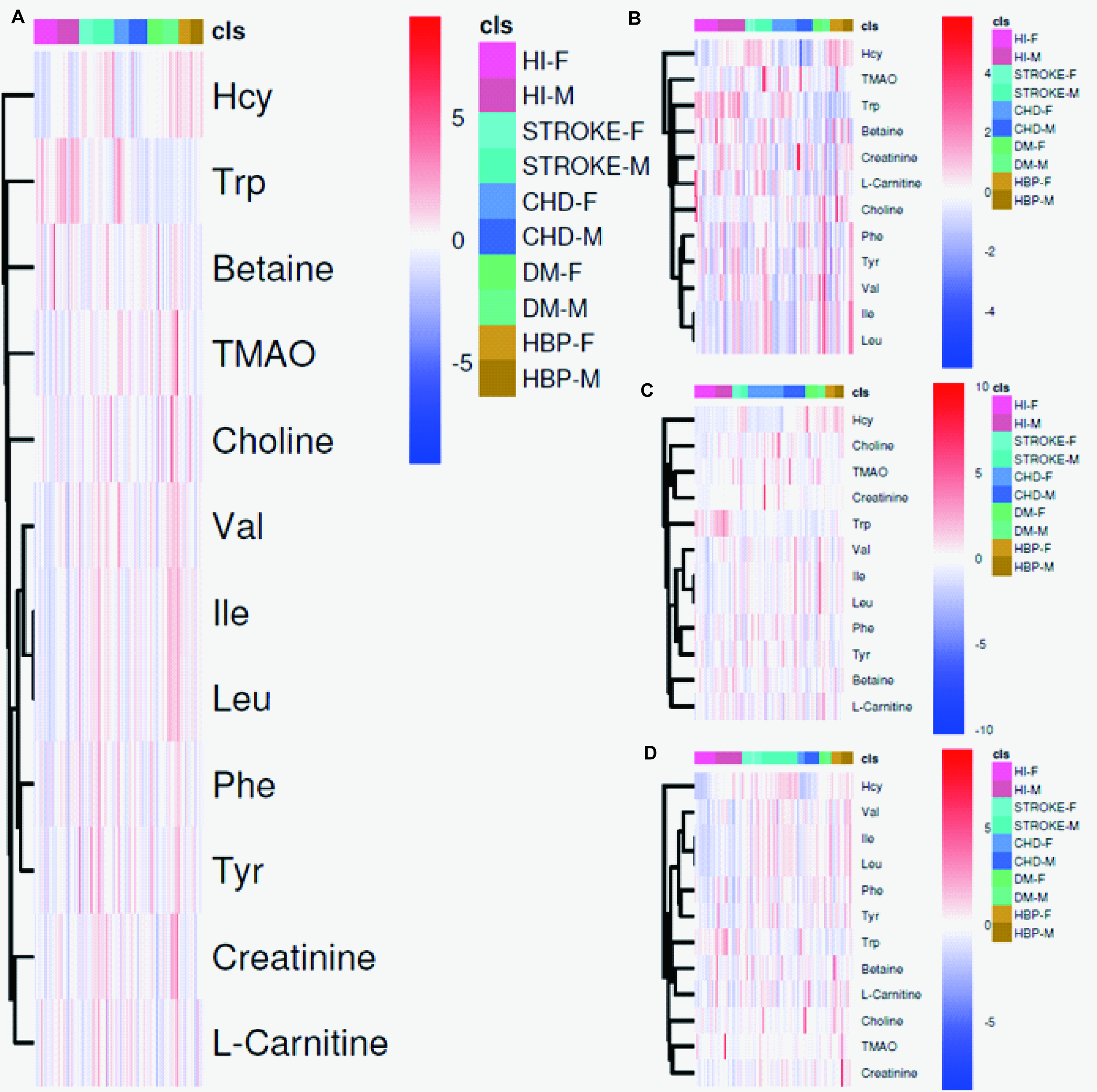
**

**Figure S1** Heat maps. A, Test Phase, B: Liaoning province, C: Ningxi province, D: Shanxi province

**
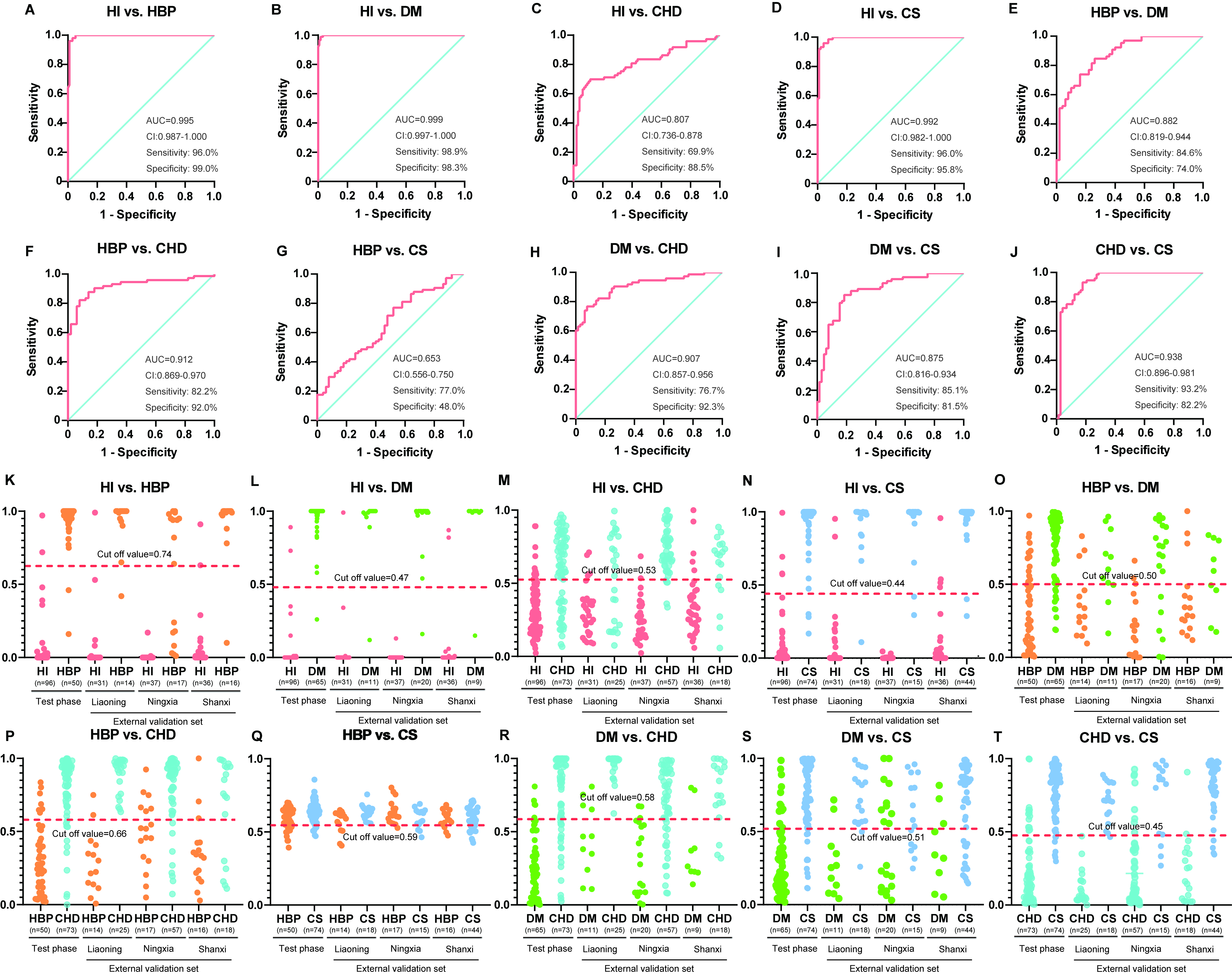
**

**Figure S2** Diagnostic outcomes and prediction accuracies without population characteristics. **A-J**: The ROC curves, on the basis of the logistic regression of each metabolite from the test set. **K-T**: The prediction accuracies by the biomarkers in the test phase and validation sets were compared between each group. HI: Healthy, DM: Diabetes, CHD: Coronary Heart Disease, HBP: High Blood Pressure

**
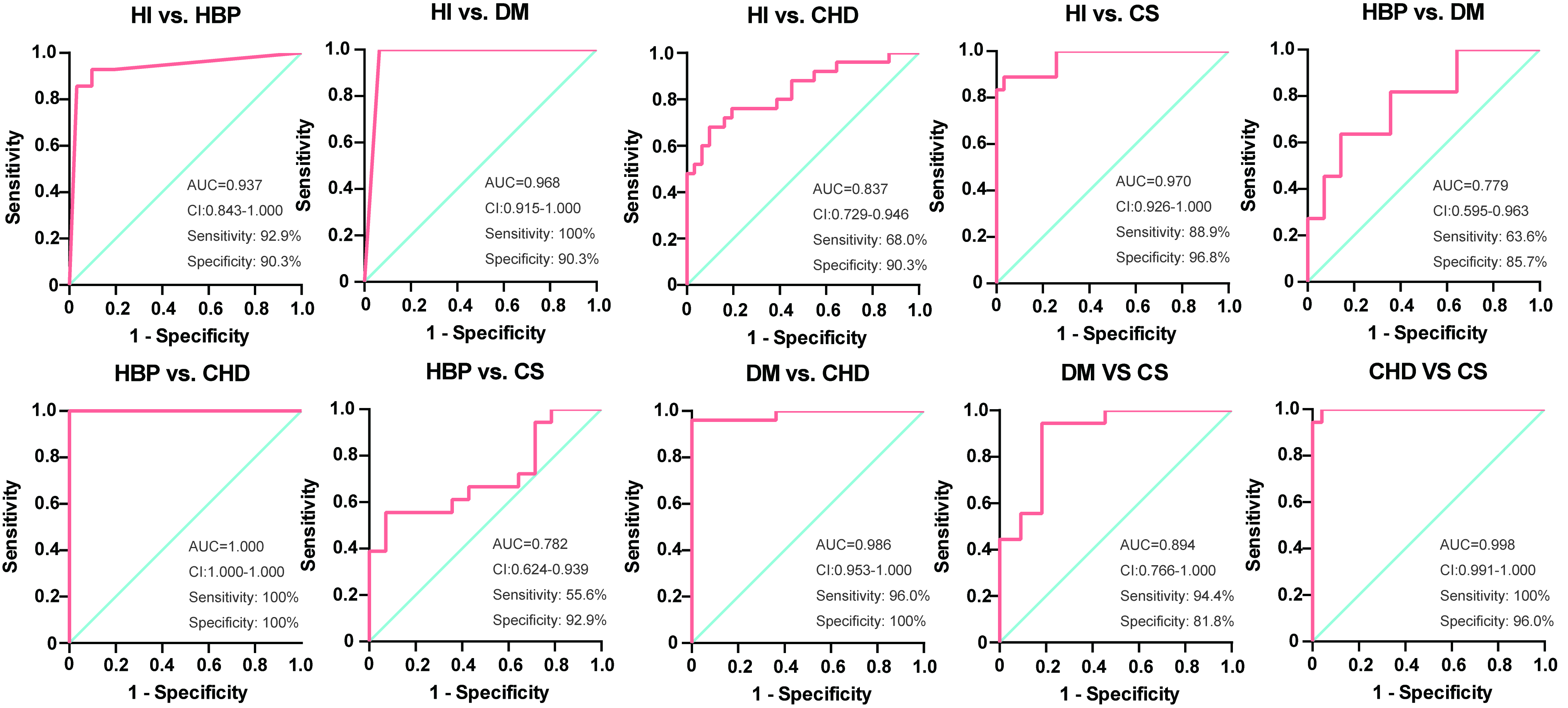
**

**Figure S3** Diagnostic outcomes by the of the biomarker panels in Liaoning province with population characteristics. HI: Healthy, DM: Diabetes, CHD: Coronary Heart Disease, HBP: High Blood Pressure

**
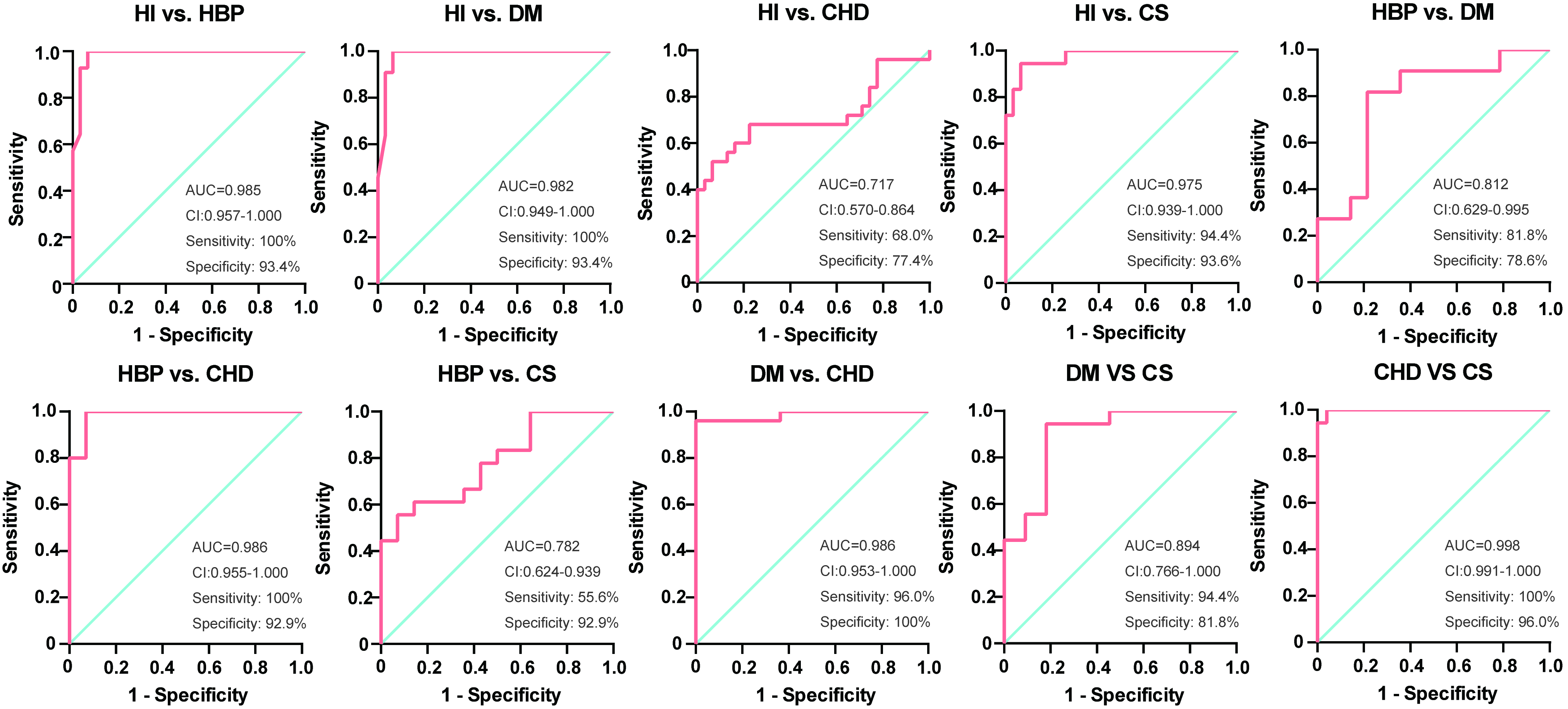
**

**Figure S4** Diagnostic outcomes by the of the biomarker panels in Liaoning province without population characteristics. HI: Healthy, DM: Diabetes, CHD: Coronary Heart Disease, HBP: High Blood Pressure

**
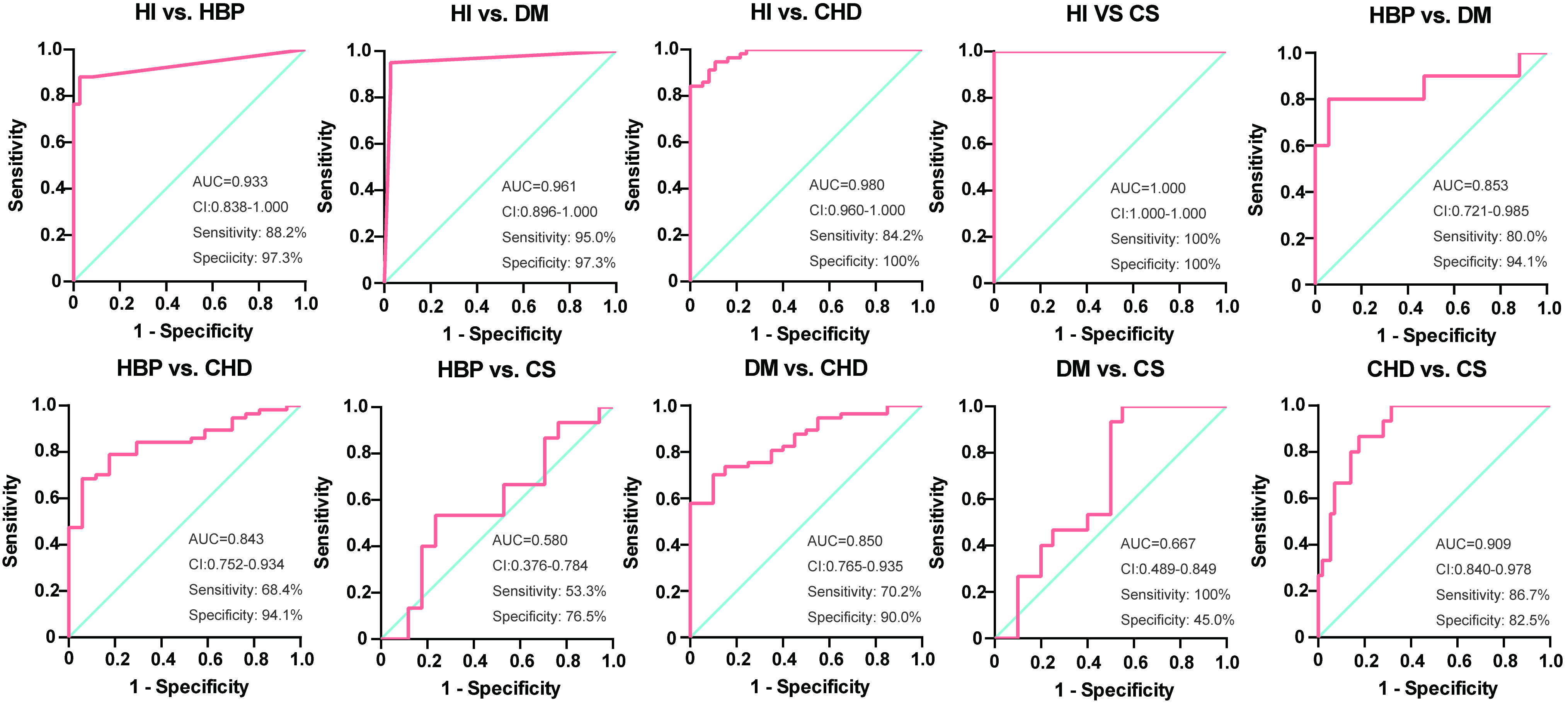
**

**Figure S5** Diagnostic outcomes by the of the biomarker panels in Ningxi province with population characteristics. HI: Healthy, DM: Diabetes, CHD: Coronary Heart Disease, HBP: High Blood Pressure

**
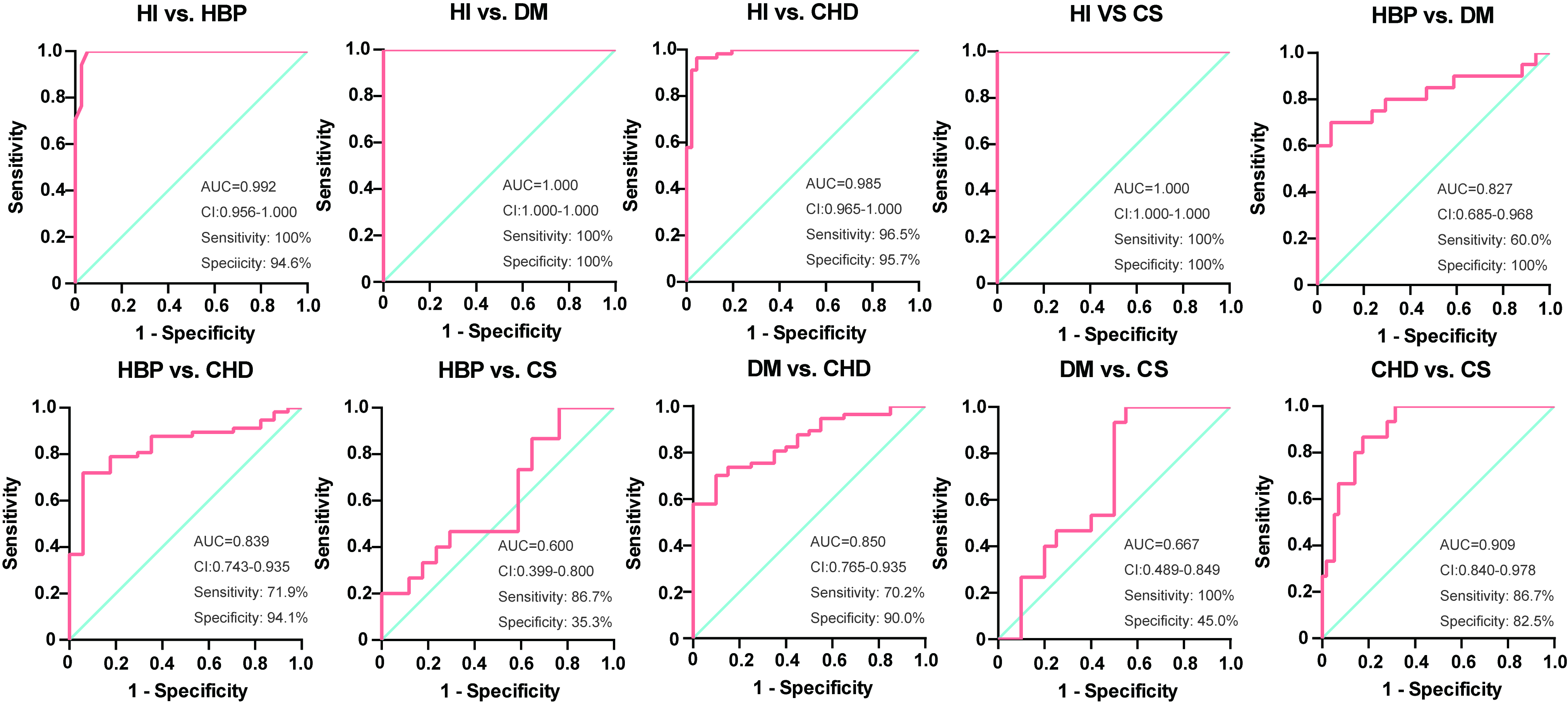
Figure S6** Diagnostic outcomes by the of the biomarker panels in Ningxi province without population characteristics. HI: Healthy, DM: Diabetes, CHD: Coronary Heart Disease, HBP: High Blood Pressure

**
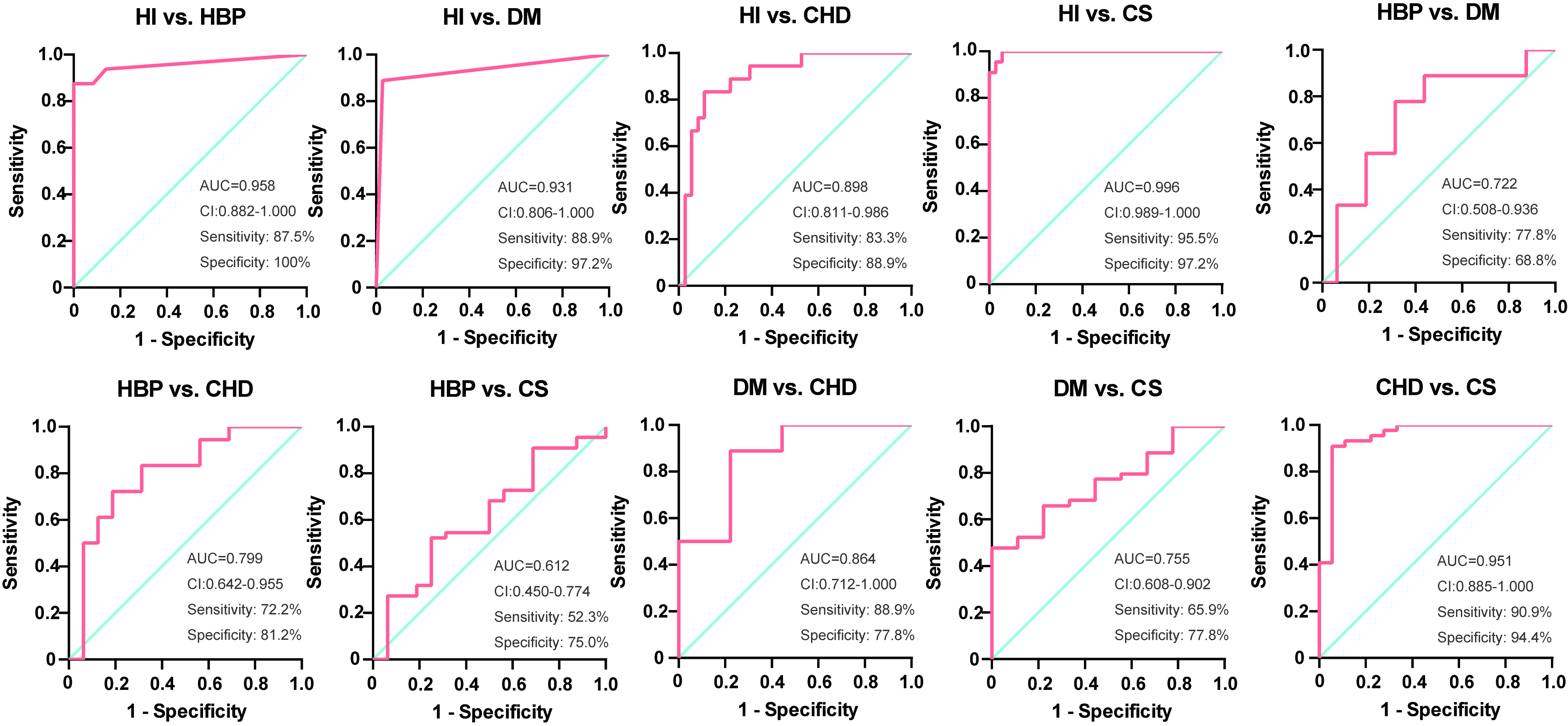
**

**Figure S7** Diagnostic outcomes by the of the biomarker panels in Shanxi province with population characteristics. HI: Healthy, DM: Diabetes, CHD: Coronary Heart Disease, HBP: High Blood Pressure

**
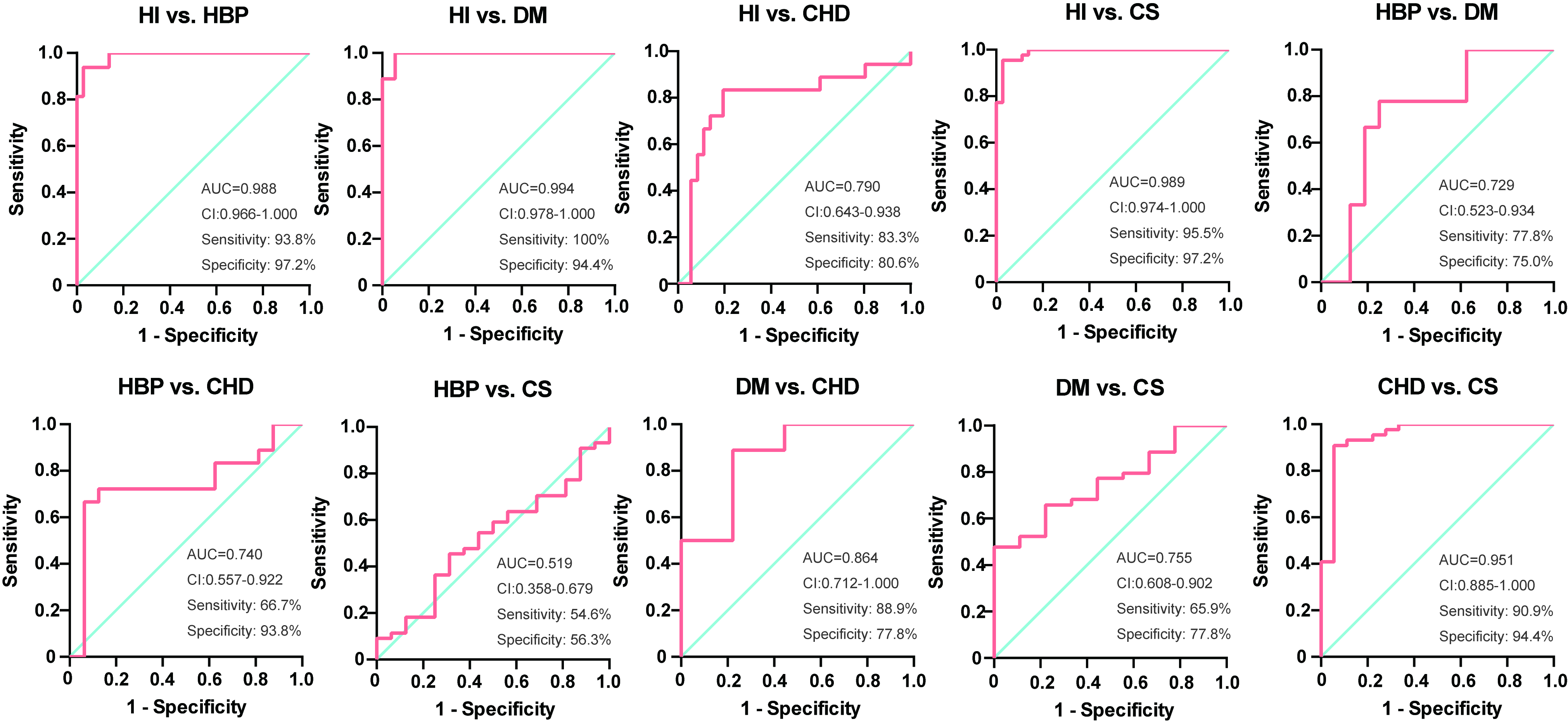
**

**Figure S8** Diagnostic outcomes by the of the biomarker panels in Shanxi province without population characteristics. HI: Healthy, DM: Diabetes, CHD: Coronary Heart Disease, HBP: High Blood Pressure

**Table S1** The main metabolites associated with CCVD

| **Disease** | **Marker** | **Reference** | **correlation with disease** |
| --- | --- | --- | --- |
| Arrhythmia | glycolithocholate sulfate | Metabolomics and Incidence of Atrial Fibrillation in African Americans: The Atherosclerosis Risk in Communities (ARIC) Study | increase |
|  | glycocholenate sulfate |  | increase |
| Atherosclerosis | cotinine | Associations Between the Serum Metabolome and All-Cause Mortality Among African Americans in the Atherosclerosis Risk in Communities (ARIC) Study | increase |
|  | mannose |  | increase |
|  | glycocholate |  | increase |
|  | pregnendioldisulfate |  | increase |
|  | α-hydroxyisovalerate |  | increase |
|  | N-acetylalanine |  | increase |
|  | andro-steroid monosulfate 2 |  | increase |
|  | uridine |  | decrease |
|  | γ-glutamyl-leucine |  | decrease |
| CAD | phytosphingosine | Comprehensive Metabolomic Characterization of Coronary Artery Diseases | increase |
|  | lysophosphatidylcholines |  | decrease |
|  | lysophosphatidylethanolamine 18:2 |  | decrease |
|  | phosphatidylethanolamine |  | decrease |
|  | monoglyceride 18:2 (MG 18:2) | Large-scale Metabolomic Profiling Identifies Novel Biomarkers for Incident Coronary Heart Disease | increase |
|  | lysophosphatidylcholine 18:2 (Lyso PC 18:2 Lyso PC 18:1) |  | increase |
|  | sphingomyelin 28:1 (SM 28:1) |  | increase |
|  | branched-chain amino acids (valine, leucine, isoleucine) | Validation of the association between a branched chain amino acid metabolite profile and extremes of coronary artery disease in patients referred for cardiac catheterization | increase |
|  | short-chain acylcarnitines |  | increase |
|  | C16:0 | Plasma Ceramides, Mediterranean Diet, and Incident Cardiovascular Disease in the PREDIMED Trial | increase |
|  | C22:0 |  | increase |
|  | C24:0 |  | increase |
|  | C24:1 ceramides |  | increase |
|  | Ceramide C24:0 |  | increase |
|  | ceramide C16:0 |  | decrease |
| Cardiovascular Disease | phenylalanine | Metabolite Profiling and Cardiovascular Event Risk: A Prospective Study of Three Population-Based Cohorts | increase |
|  | monounsaturated fatty acid |  | increase |
|  | omega-6 fatty acids |  | decrease |
|  | docosahexaenoic acid |  | decrease |
|  | Creatinine | Serum Creatinine Concentration and Risk of Cardiovascular Disease |  |
|  | Homocysteine | AACE 2017 Guidelines | increase |
|  | Uric acid |  | increase |
|  | isoleucine | A diabetes-predictive amino acid score and future cardiovascular disease | increase |
|  | tyrosine |  | increase |
|  | phenylalanine |  | increase |
| Cerebral Stroke | myristoyl-ethanolamine | Metabolomics predicts stroke recurrence after transient ischemic attack | increase |
|  | 1-monopalmitin |  | decrease |
|  | dodecanoic acid |  | decrease |
|  | meso-erythritol |  | decrease |
|  | threonate |  | decrease |
|  | lysophosphatidylcholine (LysoPC[16:0]) |  | decrease |
|  | lysophosphatidylcholine (20:4) |  | decrease |
|  | lysophosphatidylcholine (22:6) |  | decrease |
| Heart Failure | histidine | Metabolic disturbances identified in plasma are associated with outcomes in patients with heart failure diagnostic and prognostic value of metabolomics | decrease |
|  | phenylalanine |  | increase |
|  | spermidine |  | increase |
|  | phosphatidylcholine C34:4 |  | increase |
|  | methylarginine/arginine |  | increase |
|  | butyrylcarnitine |  | increase |
|  | phenylalanine | Metabolomic fingerprint of heart failure in humans: a nuclear magnetic resonance spectroscopy analysis | increase |
|  | tyrosine |  | increase |
|  | isoleucine |  | increase |
|  | creatine |  | increase |
|  | lactate |  | decrease |
|  | citrate |  | decrease |
|  | lysine |  | decrease |
|  | L-3,4-dihydroxyphenylalanine |  | decrease |
|  | lactate | Metabonomics analysis of plasma reveals the lactate to cholesterol ratio as an independent prognostic factor of short-term mortality in acute heart failure | increase |
|  | hydroxyleucine | Associations Between Metabolomic Compounds and Incident Heart Failure Among African Americans: The ARIC Study | increase |
|  | dihydroxydocosatrienoic acid |  | decrease |
|  | long-chain acylcarnitines（C16、 C18） | Prognostic Implications of Long-Chain Acylcarnitines in Heart Failure and Reversibility With Mechanical Circulatory Support | NA |
| Hypertension | oleic acid | A Targeted Metabolomics MRM-MS Study on Identifying Potential Hypertension Biomarkers in Human Plasma and Evaluating Acupuncture effects | increase |
|  | myoinositol |  | increase |
|  | serine | Identifcation of Serum Metabolites Associated With Incident Hypertension in the European Prospective Investigation into Cancer and Nutrition–Potsdam Study | increase |
|  | glycine |  | increase |
|  | acylalkyl-phosphatidylcholines C42:4 |  | increase |
|  | acylalkyl-phosphatidylcholines C44:3 |  | increase |
|  | diacyl-phosphatidylcholines C38:4 |  | increase |
|  | diacyl-phosphatidylcholines C38:3 |  | increase |
| Myocardial Infarction | arginine | Improvement of myocardial infarction risk prediction via inﬂammation-associated metabolite biomarkers | increase |
|  | LPC 17:0 |  | decrease |
|  | LPC 18:2 |  | decrease |
| Type 2 Diabetes and Cardiovascular Disease | Mannose | Plasma Mannose Levels Are Associated with Incident Type 2 Diabetes and Cardiovascular Disease | increase |

**Table S2** Population Characteristics in Communities Study of the Test Phase

|  | Healthy (n=96) | Stroke (n=74) | CHD (n=73) | Diabetes (n=65) | HBP (n=50) |
| --- | --- | --- | --- | --- | --- |
| Mean age, years (SD) | 60.7 (15.4) | 65.3 (7.0) | 67.5 (8.5) | 64.1 (6.8) | 59.1 (12.3) |
| Sex |  |  |  |  |  |
| Men | 46 (47.9%) | 43 (58.1%) | 40 (54.8%) | 33 (50.8%) | 25 (50.0%) |
| Women | 50 (52.1%) | 31 (41.9%) | 33 (45.2%) | 32 (49.2%) | 25 (50.0%) |
| Mean BMI, kg/m² (SD) | 23.0 (4.0) | 25.8 (3.9)** | 25.9 (3.7)** | 25.7 (3.6)** | 25.6 (4.1)** |
| DBP (SD) | 76.6(9.0) | 90.9 (15.4) | 90.0 (14.2) | 88.0 (14.6) | 95.4 (9.9) |
| Complication |  |  |  |  |  |
| CHD, (Unknown)^#^ | NA | 12, 16.2% (2, 2.7%) | NA | NA | NA |
| Diabetes, (Unknown)^#^ | NA | 6, 8.1% (4, 5.4%) | 3, 4.6% (4, 5.4%) | NA | NA |
| Hypertension | NA | 67 (90.5%) | 60 (82.2%) | 49 (75.4%) | NA |
| Use therapeutic drugs within a month | NA | 64 (86.5%) | 56 (76.7%) | 43 (66.2%) | 15 (30.0%) |
| Smoking |  |  |  |  |  |
| Current smoker | 35 (36.5%) | 9 (12.2%) | 18(24.7%) | 16 (24.6%) | 12 (24.0%) |
| Never smoker | 61 (63.5%) | 124 (87.8%) | 55 (75.3%) | 49 (75.4%) | 38 (76.0%) |
| Drinking | 15 (15.6%) | 4 (5.4%) | 7 (9.6%) | 6 (9.2%) | 12 (24.0%) |
| Farm work | 59 (61.5%) | 23 (31.1%) | 32(43.8%) | 25 (38.5%) | 33 (66.0%) |
| Education attainment, years (SD) | 7.0 (3.6) | 5.9 (3.3) | 5.5 (3.1) | 6.0 (3.4) | 5.2 (3.7) |

^#^ Some missing values for this category. NA=not applicable. BMI=body-mass index

**P<0.01 vs Healthy

**Table S3** Population Characteristics in Communities Study of Liaoning

|  | Healthy (n=31) | Stroke (n=18) | CHD (n=25) | Diabetes (n=11) | HBP (n=14) |
| --- | --- | --- | --- | --- | --- |
| Mean age, years (SD) | 60.4 (15.2) | 62.2 (7.4) | 67.5 (7.0) | 62.7 (10.0) | 60.9(9.3) |
| Sex |  |  |  |  |  |
| Men | 17 (54.8%) | 11 (61.1%) | 10(33.3%) | 5 (45.5%) | 6(42.9%) |
| Women | 14 (45.2%) | 7 (38.9%) | 15 (66.7%) | 6 (54.5%) | 8 (57.1%) |
| Mean BMI, kg/m² (SD) | 23.6 (3.1) | 24.7 (2.5)** | 22.6 (3.6)** | 25.7 (4.0)** | 24.3 (4.8)** |
| DBP (SD) | 78.0(7.2) | 96.1 (28.5) | 89.6 (18.3) | 87.3 (7.3) | 94.6 (16.4) |
| Complication |  |  |  |  |  |
| CHD, (Unknown)^#^ | NA | 4 (22.2%) | NA | NA | NA |
| Diabetes, (Unknown)^#^ | NA | 1 (5.5%) | 2, 5.7% | NA | NA |
| Hypertension | NA | 13 (72.2%) | 13 (52.0%) | 9 (81.8%) | NA |
| Use therapeutic drugs within a month | NA | 10 (55.5%) | 16 (64.0%) | 6(54.5%) | 2 (14.3%) |
| Smoking |  |  |  |  |  |
| Current smoker | 15 (48.4%) | 9 (50.0%) | 7(28.0%) | 2 (18.2%) | 4 (28.6%) |
| Never smoker | 16 (51.6%) | 9 (50.0%) | 18 (72.0%) | 9 (81.8%) | 10(71.4%) |
| Drinking | 6 (19.4%) | 4 (22.2%) | 4 (16.0%) | 1 (9.1%) | 4 (28.6%) |
| Farm work | 29 (93.5%) | 12 (66.7%) | 17(68%) | 9 (81.8%) | 10 (71.4%) |
| Education attainment, years (SD) | 6.4 (3.4) | 7.0 (2.5) | 5.2 (4.1) | 5.6 (3.5) | 6.1 (1.5) |

^#^ Some missing values for this category. NA=not applicable. BMI=body-mass index

**P<0.01 vs Healthy

**Table S4** Population Characteristics in Communities Study of Ningxia

|  | Healthy (n=37) | Stroke (n=15) | CHD (n=57) | Diabetes (n=20) | HBP (n=17) |
| --- | --- | --- | --- | --- | --- |
| Mean age, years (SD) | 56.4 (14.9) | 66.8 (10.8) | 62.8 (9.9) | 65.3 (6.2) | 60.0(11.6) |
| Sex |  |  |  |  |  |
| Men | 17 (45.9%) | 7 (46.7%) | 22 (38.6%) | 8 (40.0%) | 8 (47.1%) |
| Women | 20 (54.1%) | 8 (53.3%) | 35 (61.4%) | 12 (60.0%) | 9 (52.9%) |
| Mean BMI, kg/m² (SD) | 23.2 (3.1) | 26.4(3.1)** | 24.4(3.3)** | 24.0 (3.3)** | 27.5 (3.8)** |
| DBP (SD) | 76.6(9.1) | 96.7 (11.6) | 91.5 (14.3) | 90.2 (12.5) | 96.4 (12.0) |
| Complication |  |  |  |  |  |
| CHD, | NA | 6 (40%) | NA | NA | NA |
| Diabetes, (Unknown)^#^ | NA | 0 (0.0%) | 2, 3.5% (2, 3.5%) | NA | NA |
| Hypertension | NA | 14 (93.3%) | 43 (75.4%) | 16 (80.0%) | NA |
| Use therapeutic drugs within a month | NA | 13 (86.7%) | 43 (75.4%) | 13 (65.5%) | 3 (17.6%) |
| Smoking |  |  |  |  |  |
| Current smoker | 9 (24.3%) | 2(13.3%) | 7(28.0%) | 3 (15.0%) | 4 (23.5%) |
| Never smoker | 28 (75.7%) | 13 (86.7%) | 18 (72.0%) | 17 (85.0%) | 13(76.5%) |
| Drinking | 1 (2.7%) | 1 (6.7%) | 3 (5.3%) | 0 (0.0%) | 1 (5.9%) |
| Farm work | 24 (64.9%) | 5 (33.3%) | 34 (59.6%) | 13 (65.5%) | 12 (70.6%) |
| Education attainment, years (SD) | 4.5 (4.2) | 1.5 (2.7) | 2.1 (3.4) | 2.0(3.0) | 2.6 (4.1) |

^#^ Some missing values for this category. NA=not applicable. BMI=body-mass index

**P<0.01 vs Healthy

**Table S5** Population Characteristics in Communities Study of Shanxi

|  | Healthy (n=36) | Stroke (n=44) | CHD (n=18) | Diabetes (n=9) | HBP (n=16) |
| --- | --- | --- | --- | --- | --- |
| Mean age, years (SD) | 54.6 (14.7) | 63.7(8.1) | 62.4 (6.1) | 62.8 (5.8) | 57.9 (13.8) |
| Sex |  |  |  |  |  |
| Men | 17 (45.9%) | 7 (46.7%) | 22 (38.6%) | 8 (40.0%) | 8 (47.1%) |
| Women | 20 (54.1%) | 8 (53.3%) | 35 (61.4%) | 12 (60.0%) | 9 (52.9%) |
| Mean BMI, kg/m² (SD) | 23.3 (2.9) | 24.3(2.5)** | 24.7(3.1)** | 25.4(2.8)** | 24.9 (3.2)** |
| DBP (SD) | 74.8(6.6) | 85.4(13.8) | 88.4(17.0) | 86.8 (25.0) | 90.5 (16.7) |
| Complication |  |  |  |  |  |
| CHD | NA | 4 (9.1%) | NA | NA | NA |
| Diabetes, (Unknown)^#^ | NA | 0, 0.0% (1, 2.3%) | 0, 0.0% | NA | NA |
| Hypertension | NA | 39 (88.6%) | 10 (55.6%) | 7 (77.8%) | NA |
| Use therapeutic drugs within a month | NA | 33（75.0%) | 13 (72.2%) | 8 (88.9%) | 7(43.8%) |
| Smoking |  |  |  |  |  |
| Current smoker | 15 (41.7%) | 7(15.9%) | 5(27.8%) | 1 (11.1%) | 5 (31.2%) |
| Never smoker | 21 (58.3%) | 37(84.1%) | 13 (72.2%) | 8 (88.9%) | 11(68.8%) |
| Drinking | 4 (11.1%) | 3 (6.8%) | 4 (22.2%) | 0 (0.0%) | 4 (25%) |
| Farm work | 28 (75.0%) | 22 (50.0%) | 12 (66.7%) | 5 (55.6%) | 9 (56.3%) |
| Education attainment, years (SD) | 7.3(2.3) | 4.8 (3.1) | 4.9(3.9) | 5.0(1.7) | 5.2 (2.7) |

^#^ Some missing values for this category. NA=not applicable. BMI=body-mass index

**P<0.01 vs Healthy

**Table S6** The recovery of 12 compounds (n = 5)

| Biomarkers | Recovery (%) | | |
| --- | --- | --- | --- |
|  | LQC | MQC | HQC |
| Betaine | 80.2 | 92.2 | 97.3 |
| Choline | 86.9 | 91.0 | 97.7 |
| TMAO | 96.9 | 94.3 | 104.4 |
| Creatinine | 81.3 | 92.4 | 96.9 |
| L-Carnitine | 85.6 | 89.5 | 100.0 |
| Hcy | 88.2 | 85.9 | 86.6 |
| Ile | 99.0 | 95.5 | 100.4 |
| Leu | 109.8 | 100.3 | 106.1 |
| Val | 88.7 | 86.4 | 88.7 |
| Phe | 81.3 | 83.4 | 97.7 |
| Tyr | 80.0 | 88.6 | 88.0 |
| Trp | 90.7 | 87.1 | 104.7 |

Note: LQC, MQC, and HQC represent low, middle, and high concentration quality control samples, respectively

**Table S7** Intra- and interday precision, and accuracy of 12 compounds (n = 5)

| Metabolites | Betaine | Choline | TMAO | Creatinine | L-Carnitine | Hcy | Ile | Leu | Val | Phe | Tyr | Trp |
| --- | --- | --- | --- | --- | --- | --- | --- | --- | --- | --- | --- | --- |
| LQC (μmol/L) |  |  |  |  |  |  |  |  |  |  |  |  |
| Intraday |  |  |  |  |  |  |  |  |  |  |  |  |
| VE | 15.0 | 1.5 | 0.8 | 7.5 | 3.0 | 1.5 | 6.0 | 6.0 | 30.0 | 7.5 | 6.0 | 7.5 |
| VO | 14.9 | 1.5 | 0.8 | 7.4 | 2.9 | 1.5 | 5.7 | 5.8 | 28.6 | 7.3 | 6.3 | 7.8 |
| RSD | 7.1 | 5.5 | 6.0 | 6.8 | 7.8 | 7.2 | 6.2 | 8.9 | 1.7 | 4.8 | 4.7 | 4.8 |
| Accuracy | 99.3% | 102.7% | 101.3% | 98.7% | 96.7% | 102.7% | 95.0% | 96.7% | 95.3% | 97.3% | 105.0% | 104.0% |
|  |  |  |  |  |  |  |  |  |  |  |  |  |
| Interday |  |  |  |  |  |  |  |  |  |  |  |  |
| VO | 15.1 | 1.5 | 0.7 | 7.4 | 2.8 | 1.5 | 5.9 | 5.9 | 28.6 | 7.4 | 6.4 | 7.7 |
| RSD | 5.8 | 6.3 | 5.2 | 4.1 | 3.2 | 5.4 | 2.1 | 3.1 | 6.4 | 5.1 | 4.0 | 3.2 |
| Accuracy | 100.7% | 100.7% | 98.7% | 98.7% | 93.3% | 100.7% | 98.3% | 98.3% | 95.3% | 98.7% | 106.7% | 102.7% |
|  |  |  |  |  |  |  |  |  |  |  |  |  |
| MQC (μmol/L) |  |  |  |  |  |  |  |  |  |  |  |  |
| Intraday |  |  |  |  |  |  |  |  |  |  |  |  |
| VE | 50.0 | 5.0 | 2.5 | 25.0 | 10.0 | 50.0 | 20.0 | 20.0 | 80.0 | 25.0 | 20.0 | 25.0 |
| VO | 48.2 | 4.9 | 2.5 | 24.6 | 9.8 | 48.9 | 20.1 | 20.3 | 80.2 | 24.6 | 19.7 | 24.8 |
| RSD | 0.9 | 4.0 | 1.4 | 0.7 | 3.5 | 4.7 | 5.1 | 4.4 | 4.0 | 1.6 | 3.4 | 3.9 |
| Accuracy | 96.4% | 98.0% | 100.0% | 98.4% | 98.0% | 97.8% | 100.5% | 101.5% | 100.3% | 98.4% | 98.5% | 99.2% |
|  |  |  |  |  |  |  |  |  |  |  |  |  |
| Interday |  |  |  |  |  |  |  |  |  |  |  |  |
| VO | 49.6 | 5.1 | 2.4 | 25.3 | 9.9 | 49.0 | 20.2 | 20.1 | 79.2 | 24.5 | 19.6 | 24.7 |
| RSD | 3.1 | 2.6 | 2.3 | 3.9 | 5.4 | 1.6 | 5.6 | 3.9 | 4.2 | 2.8 | 3.2 | 1.5 |
| Accuracy | 99.2% | 102.0% | 96.0% | 101.2% | 99.0% | 98.0% | 101.0% | 100.5% | 99.0% | 98.0% | 98.0% | 98.8% |
|  |  |  |  |  |  |  |  |  |  |  |  |  |
| HQC (μmol/L) |  |  |  |  |  |  |  |  |  |  |  |  |
| Intraday |  |  |  |  |  |  |  |  |  |  |  |  |
| VE | 300.0 | 30.0 | 15.0 | 150.0 | 150.0 | 75.0 | 30.0 | 150.0 | 150.0 | 375.0 | 150.0 | 150.0 |
| VO | 308.5 | 31.0 | 14.6 | 148.3 | 143.2 | 73.0 | 31.1 | 156.2 | 152.0 | 381.2 | 154.1 | 147.6 |
| RSD | 1.1 | 3.9 | 1.0 | 3.5 | 1.3 | 3.6 | 4.1 | 2.8 | 2.2 | 2.0 | 5.8 | 2.9 |
| Accuracy | 102.8% | 103.3% | 97.3% | 98.9% | 95.5% | 97.3% | 103.7% | 104.1% | 101.3% | 101.7% | 102.7% | 98.4% |
|  |  |  |  |  |  |  |  |  |  |  |  |  |
| Interday |  |  |  |  |  |  |  |  |  |  |  |  |
| VO | 305.2 | 30.5 | 14.5 | 147.6 | 145.2 | 74.1 | 30.9 | 155.3 | 153.1 | 380.9 | 154.2 | 148.2 |
| RSD | 1.2 | 2.5 | 1.9 | 2.3 | 2.5 | 3.1 | 3.2 | 1.9 | 2.5 | 3.8 | 4.1 | 2.3 |
| Accuracy | 101.7% | 101.7% | 96.7% | 98.4% | 96.8% | 98.8% | 103.0% | 103.5% | 102.1% | 101.6% | 102.8% | 98.8% |

Note: LQC, MQC, and HQC represent low, middle, and high concentration quality control samples, respectively. VE, value expected; VO, value observed, RSD relative standard deviation

**Table S8** Quantitative parameters of OPLSDA

| Group | PCs | R2X | R2Y | Q2 |
| --- | --- | --- | --- | --- |
| HI vs. HBP | 2 | 47.8% | 75.8% | 72.8% |
| HI vs. Diabetes | 2 | 47.9% | 73.2% | 68.5% |
| HI vs. CHD | 2 | 42.1% | 50.2% | 44.1% |
| HI vs. Stroke | 2 | 48.0% | 76.3% | 73.8% |
| HBP vs. Diabetes | 2 | 45.3% | 40.7% | 30.3% |
| HBP vs. CHD | 1 | 11.9% | 45.5% | 36.8% |
| HBP vs. Stroke | 2 | 46.9% | 20.2% | 9.1% |
| Diabetes vs. CHD | 6 | 68.1% | 62.3% | 49.0% |
| Diabetes vs. Stroke | 3 | 48.5% | 57.1% | 48.0% |
| CHD vs. Stroke | 2 | 42.7% | 41.3% | 29.7% |

PCs: Principal Components

**Table S9** Concentrations of 12 Differential Metabolites in the Liaoning Phase

| metabolites | HI(n=31) | | |  | HBP(n=14) | | |  | Diabetes(n=11) | | |  | CHD(n=25) | | |  | Stroke(n=18) | | | p value |
| --- | --- | --- | --- | --- | --- | --- | --- | --- | --- | --- | --- | --- | --- | --- | --- | --- | --- | --- | --- | --- |
|  | Mean | ± | SD |  | Mean | ± | SD |  | Mean | ± | SD |  | Mean | ± | SD |  | Mean | ± | SD |  |
| Betaine | 88.2 | ± | 27.1 |  | 79.2 | ± | 42.1 |  | 61.2 | ± | 30.6 |  | 69.8 | ± | 23.0 |  | 71.4 | ± | 25.2 | 0.043 |
| Choline | 39.5 | ± | 36.7 |  | 43.0 | ± | 37.0 |  | 46.0 | ± | 33.0 |  | 40.1 | ± | 21.5 |  | 33.3 | ± | 13.6 | 0.825 |
| TMAO | 1.5 | ± | 0.9 |  | 1.6 | ± | 1.5 |  | 1.4 | ± | 0.8 |  | 2.4 | ± | 2.1 |  | 2.1 | ± | 2.1 | 0.148 |
| Creatinine | 51.6 | ± | 11.1 |  | 46.4 | ± | 15.6 |  | 46.0 | ± | 14.9 |  | 51.3 | ± | 20.0 |  | 50.3 | ± | 13.4 | 0.724 |
| L-Carnitine | 32.3 | ± | 10.2 |  | 29.0 | ± | 10.8 |  | 26.0 | ± | 9.8 |  | 29.0 | ± | 7.7 |  | 30.7 | ± | 8.9 | 0.362 |
| Hcy | 20.6 | ± | 2.4 |  | 25.6 | ± | 2.1 |  | 22.2 | ± | 2.5 |  | 17.4 | ± | 4.2 |  | 24.5 | ± | 1.7 | <0.001 |
| Ile | 28.1 | ± | 8.0 |  | 34.2 | ± | 11.6 |  | 36.7 | ± | 13.6 |  | 28.2 | ± | 9.4 |  | 35.8 | ± | 8.6 | 0.010 |
| Val | 159.3 | ± | 36.6 |  | 149.8 | ± | 44.6 |  | 175.2 | ± | 71.6 |  | 144.1 | ± | 42.4 |  | 161.7 | ± | 36.8 | 0.334 |
| Leu | 97.6 | ± | 26.0 |  | 114.2 | ± | 37.7 |  | 122.5 | ± | 43.8 |  | 95.9 | ± | 30.9 |  | 119.9 | ± | 28.0 | 0.021 |
| Phe | 54.1 | ± | 14.0 |  | 47.2 | ± | 15.7 |  | 51.7 | ± | 18.1 |  | 52.4 | ± | 14.2 |  | 52.3 | ± | 12.6 | 0.701 |
| Tyr | 48.1 | ± | 13.4 |  | 45.3 | ± | 16.4 |  | 45.3 | ± | 16.0 |  | 43.0 | ± | 11.8 |  | 46.0 | ± | 12.3 | 0.740 |
| Trp | 45.6 | ± | 10.7 |  | 21.8 | ± | 7.6 |  | 23.5 | ± | 7.5 |  | 36.4 | ± | 10.6 |  | 29.2 | ± | 5.4 | <0.001 |

**Table S10** Concentrations of 12 Differential Metabolites in the Ningxia Phase

| metabolites | HI(n=37) | | |  | HBP(n=17) | | |  | Diabetes(n=20) | | |  | CHD(n=57) | | |  | Stroke(n=15) | | | p value |
| --- | --- | --- | --- | --- | --- | --- | --- | --- | --- | --- | --- | --- | --- | --- | --- | --- | --- | --- | --- | --- |
|  | Mean | ± | SD |  | Mean | ± | SD |  | Mean | ± | SD |  | Mean | ± | SD |  | Mean | ± | SD |  |
| Betaine | 84.3 | ± | 35.9 |  | 76.0 | ± | 32.8 |  | 75.5 | ± | 29.3 |  | 75.1 | ± | 24.7 |  | 77.8 | ± | 39.7 | 0.692 |
| Choline | 23.3 | ± | 7.6 |  | 26.7 | ± | 8.8 |  | 26.2 | ± | 12.4 |  | 31.1 | ± | 14.1 |  | 23.4 | ± | 9.5 | 0.016 |
| TMAO | 1.3 | ± | 0.7 |  | 1.3 | ± | 0.8 |  | 2.4 | ± | 2.3 |  | 2.1 | ± | 2.3 |  | 2.4 | ± | 2.3 | 0.089 |
| Creatinine | 48.8 | ± | 11.3 |  | 55.5 | ± | 15.8 |  | 53.9 | ± | 18.6 |  | 68.4 | ± | 63.0 |  | 58.1 | ± | 33.9 | 0.255 |
| L-Carnitine | 29.2 | ± | 7.1 |  | 28.5 | ± | 8.3 |  | 32.3 | ± | 12.8 |  | 29.6 | ± | 8.6 |  | 29.3 | ± | 9.7 | 0.718 |
| Hcy | 17.2 | ± | 1.4 |  | 25.7 | ± | 3.8 |  | 25.5 | ± | 6.4 |  | 18.2 | ± | 3.6 |  | 25.7 | ± | 3.3 | <0.001 |
| Ile | 25.9 | ± | 6.5 |  | 33.3 | ± | 8.4 |  | 40.1 | ± | 16.9 |  | 33.1 | ± | 8.8 |  | 35.2 | ± | 10.7 | <0.001 |
| Val | 160.3 | ± | 38.1 |  | 170.2 | ± | 42.1 |  | 184.4 | ± | 58.9 |  | 176.7 | ± | 42.5 |  | 169.5 | ± | 48.0 | 0.312 |
| Leu | 90.9 | ± | 21.4 |  | 111.4 | ± | 27.2 |  | 132.9 | ± | 54.2 |  | 111.5 | ± | 28.8 |  | 117.9 | ± | 34.9 | <0.001 |
| Phe | 51.5 | ± | 11.5 |  | 50.7 | ± | 10.5 |  | 53.1 | ± | 14.3 |  | 57.1 | ± | 13.7 |  | 54.3 | ± | 19.0 | 0.269 |
| Tyr | 45.1 | ± | 11.5 |  | 48.0 | ± | 14.0 |  | 48.4 | ± | 12.6 |  | 52.0 | ± | 15.1 |  | 51.3 | ± | 20.1 | 0.228 |
| Trp | 48.0 | ± | 13.8 |  | 30.2 | ± | 8.8 |  | 23.2 | ± | 6.6 |  | 26.6 | ± | 6.7 |  | 25.1 | ± | 7.5 | <0.001 |

**Table S11** Concentrations of 12 Differential Metabolites in the Shanxi Phase

| metabolites | HI(n=36) | | |  | HBP(n=16) | | |  | Diabetes(n=9) | | |  | CHD(n=18) | | |  | Stroke(n=44) | | | p value |
| --- | --- | --- | --- | --- | --- | --- | --- | --- | --- | --- | --- | --- | --- | --- | --- | --- | --- | --- | --- | --- |
|  | Mean | ± | SD |  | Mean | ± | SD |  | Mean | ± | SD |  | Mean | ± | SD |  | Mean | ± | SD |  |
| Betaine | 78.9 | ± | 44.8 |  | 90.5 | ± | 49.9 |  | 80.2 | ± | 21.6 |  | 82.4 | ± | 30.0 |  | 88.7 | ± | 43.4 | 0.820 |
| Choline | 25.0 | ± | 16.4 |  | 38.1 | ± | 23.3 |  | 21.5 | ± | 4.3 |  | 36.1 | ± | 54.0 |  | 29.6 | ± | 19.9 | 0.339 |
| TMAO | 1.5 | ± | 3.5 |  | 2.0 | ± | 2.4 |  | 1.6 | ± | 1.4 |  | 1.0 | ± | 0.6 |  | 1.4 | ± | 0.8 | 0.762 |
| Creatinine | 42.6 | ± | 14.3 |  | 66.5 | ± | 55.8 |  | 51.5 | ± | 12.7 |  | 48.9 | ± | 19.9 |  | 53.7 | ± | 18.6 | 0.039 |
| L-Carnitine | 25.9 | ± | 10.5 |  | 32.4 | ± | 10.9 |  | 26.4 | ± | 9.3 |  | 28.2 | ± | 12.4 |  | 28.6 | ± | 9.2 | 0.325 |
| Hcy | 21.7 | ± | 3.4 |  | 25.9 | ± | 2.1 |  | 24.2 | ± | 0.9 |  | 19.7 | ± | 3.2 |  | 26.4 | ± | 3.0 | <0.001 |
| Ile | 21.8 | ± | 5.8 |  | 34.8 | ± | 13.7 |  | 37.9 | ± | 12.0 |  | 29.7 | ± | 10.7 |  | 36.6 | ± | 10.2 | <0.001 |
| Val | 126.4 | ± | 38.0 |  | 161.7 | ± | 52.7 |  | 163.7 | ± | 42.0 |  | 147.7 | ± | 46.0 |  | 166.0 | ± | 44.5 | 0.002 |
| Leu | 75.8 | ± | 18.2 |  | 116.3 | ± | 44.5 |  | 125.9 | ± | 38.2 |  | 100.7 | ± | 34.8 |  | 122.6 | ± | 33.4 | <0.001 |
| Phe | 47.3 | ± | 17.5 |  | 52.9 | ± | 14.9 |  | 61.1 | ± | 14.8 |  | 52.0 | ± | 16.5 |  | 54.2 | ± | 14.1 | 0.134 |
| Tyr | 37.3 | ± | 15.3 |  | 47.1 | ± | 18.3 |  | 50.4 | ± | 19.8 |  | 43.9 | ± | 14.4 |  | 48.8 | ± | 15.6 | 0.021 |
| Trp | 35.5 | ± | 12.2 |  | 22.6 | ± | 7.6 |  | 22.2 | ± | 6.9 |  | 27.8 | ± | 12.4 |  | 25.0 | ± | 7.2 | <0.001 |

**Table S12** Prediction equation between groups with population characteristics

| **Metabolites** | **Equation** | **Cut off value** |
| --- | --- | --- |
| HI VS HBP | $\frac{1}{1+e^{-(-26.857+0.405DBP-0.320H\mathrm{cy}-0.841T\mathrm{rp}+0.271Leu)}}$ | 0.63 |
| HI VS diabetes | $\frac{1}{1+e^{-(-35.641-13.376Trp+1.893Leu-9.272Hcy+0.909Val+3.657DBP)}}$ | 0.50 |
| HI vs CHD | $\frac{1}{1+e^{-(-15.511-0.119Trp+0.042Leu+0.748TMAO+0.156DBP+0.078BMI)}}$ | 0.50 |
| HI VS stroke | $\frac{1}{1+e^{-(-38.449+0.578H\mathrm{cy}+0.203Leu-0.476Trp+0.248DBP)}}$ | 0.27 |
| HBP VS diabetes | $\frac{1}{1+e^{-(2.83-0.24Trp-0.15H\mathrm{cy}-0.09MAO+0.03Val-0.0063Cre+0.06age-0.03DBP)}}$ | 0.55 |
| HBP VS CHD | $\frac{1}{1+e^{-(2.104+0.056age-0.274H\mathrm{cy}-0.03Leu+0.067Trp+0.048Creatinine)}}$ | 0.57 |
| HBP VS stroke | $\frac{1}{1+e^{-(-7.194+0.023Cre+0.093Trp+0.023Carnitine-0.011Val+0.027+Carnitin+0.077age)}}$ | 0.58 |
| diabetes VS CHD | $\frac{1}{1+e^{-(5.303+0.235Trp-0.179Hcy-0.006Leu-0.035V\mathrm{al})}}$ | 0.58 |
| diabetes VS stroke | $\frac{1}{1+e^{-(-16.45+0.431H\mathrm{cy}+0.251Trp-0.007C\mathrm{arnitine})}}$ | 0.51 |
| CHD VS stroke | $\frac{1}{1+e^{-(-12.717+0.434H\mathrm{cy}+0.026Leu)}}$ | 0.45 |

**Table S13** Prediction equation between groups without population characteristics

| **Metabolites** | **Equation** | **Cut off value** |
| --- | --- | --- |
| HI VS HBP | $\frac{1}{1+e^{-(-5.686-0.150H\mathrm{cy}-0.532T\mathrm{rp}+0.174Leu)}}$ | 0.74 |
| HI VS diabetes | $\frac{1}{1+e^{-(-1.264-0.800Trp+0.195Leu+0.008Hcy+0.017Val)}}$ | 0.47 |
| HI vs CHD | $\frac{1}{1+e^{-(0.010-0.083Trp+0.022Leu+0.493TMAO)}}$ | 0.53 |
| HI VS stroke | $\frac{1}{1+e^{-(-16.272+0.521H\mathrm{cy}+0.142Leu-0.311Trp)}}$ | 0.44 |
| HBP VS diabetes | $\frac{1}{1+e^{-(10.85-0.328Trp-0.403H\mathrm{cy}+0.026TMAO+0.032Val+0.023Creatinine)}}$ | 0.50 |
| HBP VS CHD | $\frac{1}{1+e^{-(5.502-0.276H\mathrm{cy}-0.034Leu+0.065Trp+0.060Creatinine)}}$ | 0.66 |
| HBP VS stroke | $\frac{1}{1+e^{-(0.655+0.003Creatinine+0.039Trp+0.011C\mathrm{arnitine}-0.002Val)}}$ | 0.59 |
| diabetes VS CHD* | $\frac{1}{1+e^{-(5.303+0.235Trp-0.179H\mathrm{cy}-0.006\mathrm{Leu}-0.035V\mathrm{al})}}$ | 0.58 |
| diabetes VS stroke* | $\frac{1}{1+e^{-(-16.45+0.431H\mathrm{cy}+0.251\mathrm{Trp}-0.007C\mathrm{arnitine})}}$ | 0.51 |
| CHD VS stroke* | $\frac{1}{1+e^{-(-12.717+0.434H\mathrm{cy}+0.026Leu)}}$ | 0.45 |

*As same as result with population characteristics

**Table S14** Diagnostic test evaluation index of our model in the Test Phase without population characteristics

| Group | Sensibility | Specificity | Accuracy | TPF | FPF | PPV | NPV |
| --- | --- | --- | --- | --- | --- | --- | --- |
| HI vs. HBP | 96.0% | 99.0% | 97.9% | 1.0% | 4.0% | 98.0% | 98.0% |
| HI vs. Diabetes | 98.1% | 98.3% | 98.1% | 1.7% | 1.9% | 97.0% | 98.9% |
| HI vs. CHD | 69.9% | 85.5% | 80.5% | 14.5% | 30.1% | 82.3% | 79.4% |
| HI vs. Stroke | 96.0% | 95.8% | 95.8% | 4.2% | 4.0% | 94.7% | 96.8% |
| HBP vs. Diabetes | 84.6% | 74.0% | 80.9% | 26.0% | 15.4% | 84.1% | 76.9% |
| HBP vs. CHD | 82.2% | 92.0% | 81.3% | 8.0% | 17.8% | 94.6% | 70.1% |
| HBP vs. Stroke | 77.0% | 48.0% | 64.5% | 52.0% | 23.0% | 68.3% | 57.1% |
| Diabetes vs. CHD* | 76.7% | 92.3% | 84.1% | 7.7% | 23.3% | 91.8% | 77.9% |
| Diabetes vs. Stroke* | 85.1% | 81.5% | 83.5% | 18.5% | 14.9% | 84.0% | 82.8% |
| CHD vs. Stroke* | 93.2% | 82.2% | 87.8% | 17.8% | 6.8% | 84.1% | 92.3% |

TPF: true positive fraction; FPF: false positive fraction; PPV: positive predictive value; NPV: negative predictive value

HI: Healthy individuals, CHD: Coronary Heart Disease, HBP: High Blood Pressure

*As same as result with population characteristics

**Table S15** Diagnostic test evaluation index of our model in Liaoning with population characteristics

| Group | Sensibility | Specificity | Accuracy | TPF | FPF | PPV | NPV |
| --- | --- | --- | --- | --- | --- | --- | --- |
| HI vs. HBP | 92.9% | 90.3% | 92.9% | 9.7% | 7.1% | 78.6% | 100% |
| HI vs. Diabetes | 100.0% | 90.3% | 97.6% | 9.7% | 0.0% | 91.7% | 100.0% |
| HI vs. CHD | 68.0% | 90.3% | 78.5% | 9.7% | 32.0% | 78.2% | 78.8% |
| HI vs. Stroke | 88.9% | 96.8% | 91.8% | 3.2% | 11.1% | 88.9% | 93.5% |
| HBP vs. Diabetes | 63.6% | 85.7% | 68.0% | 14.3% | 36.4% | 63.6% | 71.4% |
| HBP vs. CHD | 100.0% | 100% | 100% | 0.0% | 0.0% | 100% | 100.0% |
| HBP vs. Stroke | 55.6% | 92.9% | 62.5% | 7.1% | 44.4% | 66.0% | 57.1% |
| Diabetes vs. CHD | 96.0% | 100% | 88.9% | 0% | 4.0% | 86.2% | 100.0% |
| Diabetes vs. Stroke | 94.4% | 81.8% | 86.2% | 18.2% | 5.6% | 88.9% | 81.8% |
| CHD vs. Stroke | 100.0% | 96.0% | 97.7% | 4.0% | 0.0% | 94.7% | 100.0% |

TPF:true positive fraction; FPF:false positive fraction; PPV: positive predictive value; NPV: negative predictive value

HI: Healthy individuals; CHD: Coronary Heart Disease; HBP: High Blood Pressure

**Table S16** Diagnostic test evaluation index of our model in Liaoning without population characteristics

| Group | Sensibility | Specificity | Accuracy | TPF | FPF | PPV | NPV |
| --- | --- | --- | --- | --- | --- | --- | --- |
| HI vs. HBP | 100.0% | 93.4% | 93.3% | 6.6% | 0.0% | 92.3% | 93.8% |
| HI vs. Diabetes | 100.0% | 93.4% | 95.2% | 3.2% | 0.0% | 90.9% | 96.8% |
| HI vs. CHD | 68.0% | 77.4% | 22.6% | 19.4% | 32.0% | 77.8% | 71.1% |
| HI vs. Stroke | 100.0% | 100.0% | 93.9% | 0.0% | 0.0% | 89.5% | 96.7% |
| HBP vs. Diabetes | 63.6% | 78.6% | 80.0% | 21.4% | 36.4% | 75.0% | 84.6% |
| HBP vs. CHD | 100.0% | 92.9% | 84.6% | 7.1% | 0.0% | 95.2% | 72.2% |
| HBP vs. Stroke | 16.7% | 92.9% | 65.6% | 7.1% | 83.3% | 65.2% | 66.7% |
| Diabetes vs. CHD* | 100.0% | 72.7% | 88.9% | 27.3% | 0.0% | 86.2% | 100.0% |
| Diabetes vs. Stroke* | 88.9% | 81.8% | 86.2% | 18.2% | 11.1% | 88.9% | 81.8% |
| CHD vs. Stroke* | 100.0% | 100.0% | 97.7% | 0.0% | 0.0% | 94.7% | 100.0% |

TPF: true positive fraction; FPF: false positive fraction; PPV: positive predictive value; NPV: negative predictive value

HI: Healthy individuals; CHD: Coronary Heart Disease; HBP: High Blood Pressure

*As same as result with population characteristics

**Table S17** Diagnostic test evaluation index of our model in Ningxia with population characteristics

| Group | Sensibility | Specificity | Accuracy | TPF | FPF | PPV | NPV |
| --- | --- | --- | --- | --- | --- | --- | --- |
| HI vs. HBP | 88.2% | 97.3% | 96.3% | 2.7% | 11.8% | 94.1% | 97.3% |
| HI vs. Diabetes | 95.0% | 97.3% | 98.2% | 2.7% | 5.0% | 100.0% | 97.4% |
| HI vs. CHD | 84.2% | 100.0% | 88.3% | 0.0% | 15.8% | 94.2% | 81.0% |
| HI vs. Stroke | 100.0% | 100.0% | 100.0% | 0.0% | 0.0% | 100.0% | 100.0% |
| HBP vs. Diabetes | 80.0% | 94.1% | 78.4% | 5.9% | 20.0% | 92.9% | 69.6% |
| HBP vs. CHD | 68.4% | 94.1% | 79.7% | 5.9% | 31.6% | 90.4% | 54.5% |
| HBP vs. Stroke | 53.3% | 76.5% | 50.0% | 23.5% | 46.7% | 47.1% | 53.3% |
| Diabetes vs. CHD | 70.2% | 90.0% | 76.6% | 10.0% | 29.8% | 93.3% | 53.1% |
| Diabetes vs. Stroke | 100% | 45.0% | 54.3% | 55.0% | 0.0% | 47.4% | 62.5% |
| CHD vs. Stroke | 86.7% | 82.5% | 84.7% | 17.5% | 13.3% | 60.0% | 94.2% |

TPF:true positive fraction; FPF:false positive fraction; PPV: positive predictive value; NPV: negative predictive value

HI: Healthy individuals; CHD: Coronary Heart Disease; HBP: High Blood Pressure

**Table S18** Diagnostic test evaluation index of our model in Ningxia without population characteristics

| Group | Sensibility | Specificity | Accuracy | TPF | FPF | PPV | NPV |
| --- | --- | --- | --- | --- | --- | --- | --- |
| HI vs. HBP | 100.0% | 93.4% | 85.7% | 6.6% | 0.0% | 100.0% | 82.2% |
| HI vs. Diabetes | 100.0% | 93.4% | 98.2% | 6.6% | 5.0% | 100.0% | 97.4% |
| HI vs. CHD | 68.0% | 77.4% | 92.6% | 32.0% | 22.6% | 96.3% | 87.5% |
| HI vs. Stroke | 94.4% | 93.6% | 96.2% | 6.4% | 5.6% | 100.0% | 94.9% |
| HBP vs. Diabetes | 81.8% | 78.6% | 75.7% | 21.4% | 18.2% | 82.4% | 70.0% |
| HBP vs. CHD | 100% | 92.9% | 74.3% | 0.0% | 7.1% | 93.2% | 46.7% |
| HBP vs. Stroke | 55.6% | 92.9% | 40.6% | 44.4% | 7.1% | 40.0% | 41.7% |
| Diabetes vs. CHD* | 70.2% | 90.0% | 76.6% | 10.0% | 29.8% | 93.3% | 53.1% |
| Diabetes vs. Stroke* | 100% | 45.0% | 54.3% | 55.0% | 0.0% | 47.4% | 62.5% |
| CHD vs. Stroke* | 86.7% | 82.5% | 84.7% | 17.5% | 13.3% | 60.0% | 94.2% |

TPF: true positive fraction; FPF: false positive fraction; PPV: positive predictive value; NPV: negative predictive value

HI: Healthy individuals; CHD: Coronary Heart Disease; HBP: High Blood Pressure

*As same as result with population characteristics

**Table S19** Diagnostic test evaluation index of our model in Shanxi with population characteristics

| Group | Sensibility | Specificity | Accuracy | TPF | FPF | PPV | NPV |
| --- | --- | --- | --- | --- | --- | --- | --- |
| HI vs. HBP | 87.5% | 100.0% | 96.2% | 12.5% | 0.0% | 93.8% | 97.2% |
| HI vs. Diabetes | 88.9% | 97.2% | 95.6% | 2.8% | 11.1% | 88.9% | 97.3% |
| HI vs. CHD | 83.3% | 88.9% | 83.3% | 11.1% | 16.7% | 80.0% | 84.6% |
| HI vs. Stroke | 95.5% | 97.2% | 97.5% | 2.8% | 4.5% | 95.7% | 100.0% |
| HBP vs. Diabetes | 77.8% | 66.8% | 64.0% | 22.2% | 33.2% | 50% | 73.3% |
| HBP vs. CHD | 72.2% | 81.2% | 73.5% | 18.8% | 27.8% | 80.0% | 68.4% |
| HBP vs. Stroke | 52.3% | 75.0% | 56.7% | 25.0% | 47.7% | 82.1% | 34.4% |
| Diabetes vs. CHD | 88.9% | 77.8% | 82.1% | 22.2% | 11.1% | 88.9% | 70.0% |
| Diabetes vs. Stroke | 65.9% | 77.8% | 67.9% | 22.2% | 34.1% | 90.9% | 30.0% |
| CHD vs. Stroke | 90.9% | 94.4% | 90.3% | 9.1% | 5.6% | 95.2% | 80.0% |

TPF:true positive fraction; FPF:false positive fraction; PPV: positive predictive value; NPV: negative predictive value

HI: Healthy individuals; CHD: Coronary Heart Disease; HBP: High Blood Pressure

**Table S20** Diagnostic test evaluation index of our model in Shanxi without population characteristics

| Group | Sensibility | Specificity | Accuracy | TPF | FPF | PPV | NPV |
| --- | --- | --- | --- | --- | --- | --- | --- |
| HI vs. HBP | 93.8% | 97.2% | 96.2% | 2.8% | 6.2% | 93.8% | 97.2% |
| HI vs. Diabetes | 100% | 94.4% | 93.3% | 5.6% | 0.0% | 80% | 97.3% |
| HI vs. CHD | 83.3% | 80.6% | 81.5% | 19.4% | 16.7% | 72.2% | 86.1% |
| HI vs. Stroke | 95.5% | 97.2% | 92.5% | 2.8% | 4.5% | 91.3% | 94.1% |
| HBP vs. Diabetes | 77.8% | 75.0% | 76.0% | 22.2% | 25% | 66.7% | 81.3% |
| HBP vs. CHD | 66.7% | 93.8% | 76.5% | 6.2% | 33.3% | 91.7% | 68.2% |
| HBP vs. Stroke | 54.6% | 56.3% | 55.0% | 43.7% | 45.4% | 77.4% | 31.0% |
| Diabetes vs. CHD* | 88.9% | 77.8% | 82.1% | 22.2% | 11.1% | 88.9% | 70.0% |
| Diabetes vs. Stroke* | 65.9% | 77.8% | 67.9% | 22.2% | 34.1% | 90.9% | 30.0% |
| CHD vs. Stroke* | 90.9% | 94.4% | 90.3% | 0.0% | 0.0% | 95.2% | 80.0% |

TPF: true positive fraction; FPF: false positive fraction; PPV: positive predictive value; NPV: negative predictive value

HI: Healthy individuals; CHD: Coronary Heart Disease; HBP: High Blood Pressure

*As same as result with population characteristics
